# Impact of the COVID-19 pandemic on services for patients with chronic kidney disease: findings of a national survey of UK kidney centres

**DOI:** 10.1186/s12882-023-03344-6

**Published:** 2023-12-04

**Authors:** Lucy Mackintosh, Amanda Busby, Ken Farrington, Janine Hawkins, Sarah Afuwape, Paul Bristow, Maria Da Silva-Gane, Natalie Hall, Tess Harris, Joanna Hudson, Sam Norton, Paula Ormandy, Christina J. Pearce, Shalini Santhakumaran, Shivani Sharma, Sivakumar Sridharan, Retha Steenkamp, Julie Slevin, David Wellsted, Joseph Chilcot

**Affiliations:** 1 School of Life and Medical Sciences, University of Hertfordshire, College Lane Campus, Hatfield, AL10 9AB UK; 2 Renal Unit, Lister Hospital, Stevenage, SG1 4AB UK; 3 Nephrology, Urology and Renal Transplant, Royal Free London NHS Foundation Trust, Royal Free Hospital, Pond Street, London, NW3 2QG UK; 4 Department of Renal Medicine, UCL Medical School, Rowland Hill Street, London, NW3 2PF UK; 5 Kidney Care UK, Alton, GU34 1EF UK; 6 The Polycystic Kidney Disease Charity, 91 Royal College St, London, NW1 0SE UK; 7 Health Psychology Section, Department of Psychology, Institute of Psychiatry, Psychology & Neuroscience, King’s College London, 5th Floor Bermondsey Wing, Guy’s Campus, London Bridge, London, SE1 9RT UK; 8 School of Health and Society, University of Salford, Salford, M6 6PU UK; 9 The UK Kidney Association, Brandon House, Building 20a1, Southmead Road, Bristol, BS34 7RR UK

**Keywords:** Coronavirus, Chronic kidney disease, Kidney care, Service provision

## Abstract

**Background:**

Services for patients with kidney disease underwent radical adaptations in response to the COVID-19 pandemic. We undertook an online national survey of UK kidney centres to understand the nature, range, and degree of variation in these changes and to explore factors contributing to differing practice.

**Methods:**

The survey was designed by a multidisciplinary team of kidney professionals, service users and researchers. It enquired about centre services and staffing, including psychosocial provision, and changes to these in response to the COVID-19 pandemic. Links to the survey were sent to all 68 UK kidney centres and remained active from December 2021 to April 2022, and a revised version to nurses in late 2022 for additional data. Quantitative data were analysed descriptively. Content analysis on free-text responses identified common themes.

**Results:**

Analysable responses were received from 41 out of the 68 UK centres (60%), with partial data from an additional 7 (11%). Adaptations were system-wide and affected all aspects of service provision. Some changes were almost universal such as virtual consultations for outpatient appointments, with significant variation in others. Outpatient activity varied from fully maintained to suspended. Many centres reduced peritoneal dialysis access provision but in some this was increased. Centres considered that changes to transplant surgical services and for patients with advanced CKD approaching end-stage kidney disease had the greatest impact on patients. Few centres implemented adjustments aimed at vulnerable and underrepresented groups, including the frail elderly, people with language and communication needs, and those with mental health needs. Communication issues were attributed to rapid evolution of the pandemic, changing planning guidance and lack of resources. Staffing shortages, involving all staff groups particularly nurses, mainly due to COVID-19 infection and redeployment, were compounded by deficiencies in staffing establishments and high vacancy levels. Centres cited three main lessons influencing future service delivery, the need for service redesign, improvements in communication, and better support for staff.

**Conclusion:**

Kidney centre responses to the pandemic involved adaptations across the whole service. Though some changes were almost universal, there was wide variation in other areas. Exploring the role of centre characteristics may help planning for potential future severe service disruptions.

**Supplementary Information:**

The online version contains supplementary material available at 10.1186/s12882-023-03344-6.

## Introduction

The COVID-19 pandemic had a seismic global impact [1, 2]. Mortality, especially in vulnerable groups, was high, and broad-sweeping, government mandated preventative measures, including lockdowns, impinged on all aspects of society. Health and social care services found themselves under unprecedented pressure, necessitating the rapid implementation of fundamental changes to service provision with the aim of concentrating resources on acutely ill patients suffering from COVID-19 infection and its complications [3–5].

Services for patients with kidney disease were particularly affected. The care required for these patients is complex and diverse. Kidney replacement therapy (KRT) – by dialysis or transplantation – is required for those with advanced chronic kidney disease (CKD). In most countries with stable healthcare infrastructure, centre-based haemodialysis (HD) is the dominant form of dialysis, requiring patients to travel, usually three times weekly to HD units for treatment. Home treatments by peritoneal dialysis (PD) and home haemodialysis (HHD) are usually carried out by the patients themselves with or without help from relatives or carers. Assisted PD, which usually involves visits by external agencies to initiate treatment sessions, is available for less able patients in many areas. Kidney transplant surgery takes place in specialised centres. Long-term follow-up is required for transplant recipients who require life-long immunosuppression to prevent rejection. Those with less advanced kidney disease require management to treat underlying disease processes, reduce disease progression and prepare for KRT. Some of these have systemic diseases affecting the kidney and also require immunosuppressive treatment. Finally, acute, potentially recoverable, kidney impairment (AKI) was a particular problem during the initial wave of the pandemic. All these aspects of kidney care were impacted to a significant but variable degree by the pandemic [6].

Within weeks of the onset of the pandemic, guidelines were published in relation to services for patients with kidney disease. In the UK, the National Institute for Health and Care Excellence (NICE) produced guidelines on the management of services in relation to AKI [7], CKD [8], dialysis [9] and transplantation [10]. These guidelines aimed to help optimise patient safety, protect staff from infection, and enable services to make best use of NHS resources. Similar guidance was available in other parts of the world.

Since then, there have been a number of reports of responses of kidney services to the pandemic. Management of COVID-19 related AKI during the first wave of the pandemic was hampered by shortages of staff, equipment, and consumables. This necessitated flexible approaches to the use of staff and to KRT delivery including changes to intermittent and continuous therapy protocols and the use of acute PD [11–13].

A systematic review [14] found global reductions in access to kidney transplantation, dialysis and in-person nephrology care, and an increased use of telemedicine. Other publications have reported on similar adaptations to the whole kidney service provision in different countries though with variations [15–19]. For transplant services Papalois et al., based on webinar discussions, described broadly similar global findings, to the aforementioned review [20]. There were comparable findings in transplant services in different national settings [16, 21–24] with some again emphasising heterogeneity in response across settings [16, 21, 24]. Aylward et al. [25], based on a web-based survey, found marked global differences in the response of centre-based HD services with differences in COVID-19 infection rates (lower rates reported in North East Asia), availability of PPE (Africa being under resourced) and service redesign (95% of centres in East & Central Europe and South Asia had dedicated HD shifts to isolate COVID-19 infected patients). Other national and single centre studies have described a similar range of adaptations in centre-based HD services [26–29]. Global surveys of PD provision have reported marked within-region and across-region variability in facility burden, clinical practice, and adaptation to the pandemic [30]. National surveys, which focussed on the impact for PD, have echoed these findings [31]. Reports of adaptations in HHD services are dominated by use of telemedicine [32]. The implications of the pandemic as a driver for expanding future use of home therapies have been highlighted [33]. Reductions in face-to-face consultations in favour of telemedicine contacts and reduced frequency of monitoring of patients with progressive CKD have also been reported [34].

The nature and range of responses of kidney centres to the problems posed by the pandemic has varied in relation to factors including location, population served, and by treatment modality. We have undertaken a national survey of UK kidney centres to understand the nature, range, and degree of variation in these responses, with particular emphasis on patients with CKD whatever their treatment modality rather than those with AKI. We also wished to explore possible reasons for variation.

## Methods

### Rationale of the survey

An online survey was devised to provide data for two National Institute for Health and Care Research (NIHR) portfolio studies:

1. *Centre characteristics, practice patterns and the experience of kidney patients during the COVID-19 pandemic –* funded by British Renal Society (now part of the UK Kidney Association) and Kidney Care UK.
2. *A national study of practice patterns in renal services in the identification and management of depression in people with chronic kidney disease –* funded by Kidney Research UK and the Stoneygate Trust.

The survey aimed to define the characteristics of UK kidney treatment centres, the services they provide, including psychological and social work provision, and the service responses during the COVID-19 pandemic. Combining the surveys aimed to reduce the research burden on the NHS staff during the Omicron COVID-19 wave (from November 2021) since there was a degree of overlap in data requirements between the studies. This report addresses the general survey findings with respect to impact of COVID-19 on services. Findings related to psychological and social work provision, will be reported elsewhere.

### Design

The survey was designed by a multi-disciplinary group with extensive experience of kidney care. It involved people living with advanced kidney disease, nurses, nephrologists, psychologists, social workers, counsellors, representatives of kidney patient charities, and academics with a background of research in this area. The survey comprised three sections: (1) Characteristics of the kidney centre including services and staffing, to be completed by the Clinical Director or Lead Nurse (*n* = 61 questions), (2) psychological support, to be completed by in-house psychological support staff (30 questions), and (3) social work support, to be completed by in-house social work staff 23 questions). The responses to the COVID-19 pandemic were enquired about in each of these areas. Sections 2 and 3 focused on the identification and management of depression and depressive symptoms. The questions were formulated as a combination of multiple choice, free text, yes/no, and rank ordering. The draft survey was piloted in two kidney units and final amendments made based on this feedback. It was estimated to take approximately 30 min to complete. The online survey was hosted via the University of Hertfordshire’s REDCap site, a secure web application. A copy of the survey can be found in Additional file 1.

### Data collection

A link to the survey was circulated to all 68 UK kidney centres in December 2021, via email. The survey remained open until 30th April 2022. Reminder emails were sent to centres monthly. The initial response from lead nurses was poorer than expected, so an abbreviated questionnaire was recirculated to lead nurses in December 2022, available until January 2023, to gather additional information. Participant information and consent were included within the survey link provided. On completion of section one (the general module) the initial respondent was required to insert the email addresses of the leads for social work and psychological provision in the centre (if applicable). The named staff members were then automatically sent a link within REDCap to complete the accompanying relevant sections of the survey. Where no additional staff names were provided, the Clinical Director was asked about any formal or informal policies they may use for the identification of psychological and social issues. Following this the survey was classed as complete.

### Data analysis

Upon survey closure, data were downloaded into a csv file and prepared for analysis. For items answered by both Clinical Directors and nurses, or other duplications, data were compared and combined where appropriate. In the case of discrepancies, the value was included according to who was deemed to have provided the most informed answer. The data was summarised using frequencies and proportions, cross tabulating questions where appropriate. All analyses were performed in Stata/IC 15.1. Some questions within the survey required a free-text response. The qualitative data were initially assessed, and content analysis performed to identify any common themes arising. Answers with similar semantic meanings were clustered together in the analysis and an appropriate code assigned to demonstrate the meaning [35]. A quality check of the codes was completed to address trustworthiness. The summative content analysis aims to provide a summary of subject matter entered to the survey.

## Results

There were 42 responses to the general module, 41 (60% of all UK centres) of which were analysable. The later abbreviated nursing questionnaire provided an additional seven novel responses, making a total response from 48 (71%) individual centres. The psychology module received 20 responses (all analysable) and the social work module 16 (13 analysable). Percentages displayed in subsequent descriptions relate to the number of responders to the particular question in relation to the number of analysable responses to the relevant section (i.e., up to 41 or 48).

Practically all responding centres provided outpatient care for patients with advanced CKD, in-centre HD, home therapies (includes both HHD and PD), transplant follow-up and conservative management (non-KRT symptom control). Most provided satellite HD, assisted PD, and transition services. Transplant surgery was provided by 17 centres (35%).

### Service adaptations during COVID-19

#### Inpatient service

Most centres (90%) had introduced ‘cohorting’ by COVID-19 status (treating patients with COVID-19 separately). 50% had instituted isolation wards. Expansion of dialysis facilities to ICU (76%) and plumbing-in additional ward beds for HD (12%) was described. These changes were introduced mainly in wave 1 of the pandemic (March 2020 to August 2020).

#### Centre and satellite-based HD (Fig. 1)

**Fig. 1:**
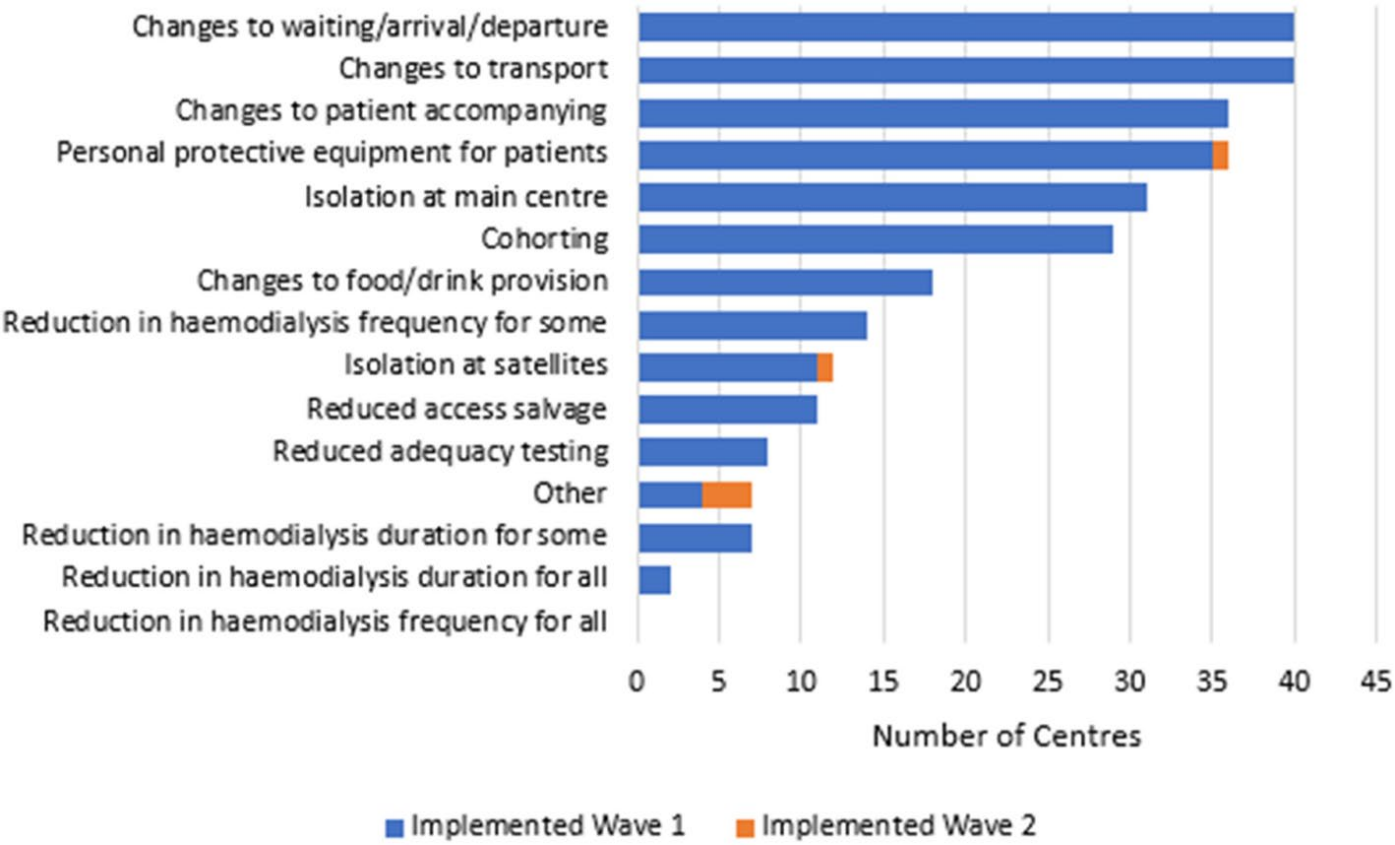
Adaptations to centre- and satellite-based haemodialysis provision, by wave of first implementation

Most introduced ‘cohorting’ or isolation areas at the centre or in satellite units. Changes to patient transport, with more focus on individual requirements, were almost universal as were changes to arrival procedures e.g., temperature check, handwashing, restriction of accompanying individuals, and provision of masks for patients. One third of units reduced dialysis frequency for some patients. One sixth reduced dialysis duration for some, and two units reduced it for all. Access salvage procedures (26%) and adequacy testing (19%) were also reduced. Changes largely were implemented in wave 1. Later a number of units introduced PCR screening.

#### Outpatient services (Fig. 2)

**Fig. 2:**
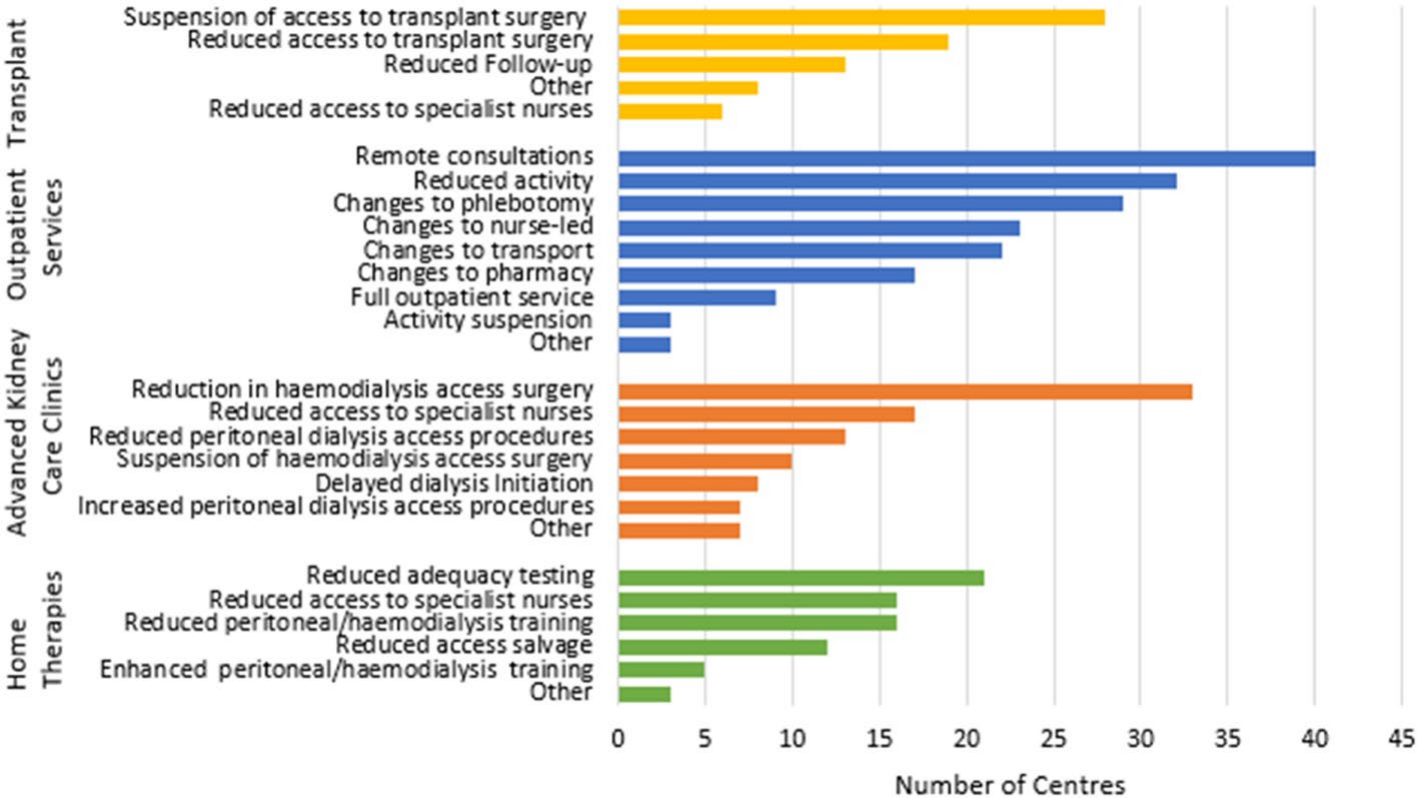
Adaptations in outpatient services, advanced kidney care clinics, home therapies and transplantation services

Changes to outpatient services were almost universal. Activity was reduced in most (78%) and completely suspended in a few (7%) of these. However, around 20% of centres managed to retain a full outpatient programme. Where activity was continued, much took place by telephone or video link. Support services were reconfigured. These included nurse-led clinics, pharmacy services, phlebotomy, and transport. Similar proportions increased and decreased such provision. In advanced kidney care (AKCC) clinics, a large proportion of centres reduced access surgery. A small proportion suspended it altogether. Transfer of access procedures to (NHS-funded) private sector was also described. In contrast, though PD access was reduced in about one third of centres, in another 17% it was increased. Over 40% of centres reported reduced access to specialist AKCC nurses. Reductions in education sessions for AKCC patients was also reported. Services for outpatients on immune suppressive agents were also disrupted with 39% of units reporting reducing access to immunosuppressive agents and 15% reduced access to specialist nurses. Most changes took place in wave 1.

#### Home therapies (Fig. 2)

Thirty-nine percent of centres reported reducing training for home therapies. In contrast, a small proportion (12%) managed to increase it. One centre switched PD training completely to the home setting and another increased both PD and HHD activity. Reduced access to specialist nursing was common. Adequacy testing and access salvage procedures were frequently reduced. Others reported increasing use of remote monitoring and speeding up transfers from centre to home.

#### Transplantation (Fig. 2)

Most centres reduced or suspended transplant surgery. Transplant follow-up was reduced, along with access to specialist nurses. Virtual follow-up and remote phlebotomy were also common. Some non-transplanting centres reported changing transplant provider to maintain some throughput.

#### Psychosocial services

Moderate to significant disruption to psychosocial services was reported, predominantly due to staff absences with COVID-19 infection and redeployment (each 30% of responding centres). 78% of lead psychologists reported a significantly increased caseload, with several noting reduced access to IAPT services (Improving Access to Psychological Therapies). Lead social workers also reported increased caseload numbers and complexity along with significant reductions in referrals to external agencies. Much psychosocial provision was carried out virtually, and on an individual rather than group basis. Other findings from the Psychosocial components of the survey will be reported elsewhere.

### Centre-assessed impact on services

**Fig. 3:**
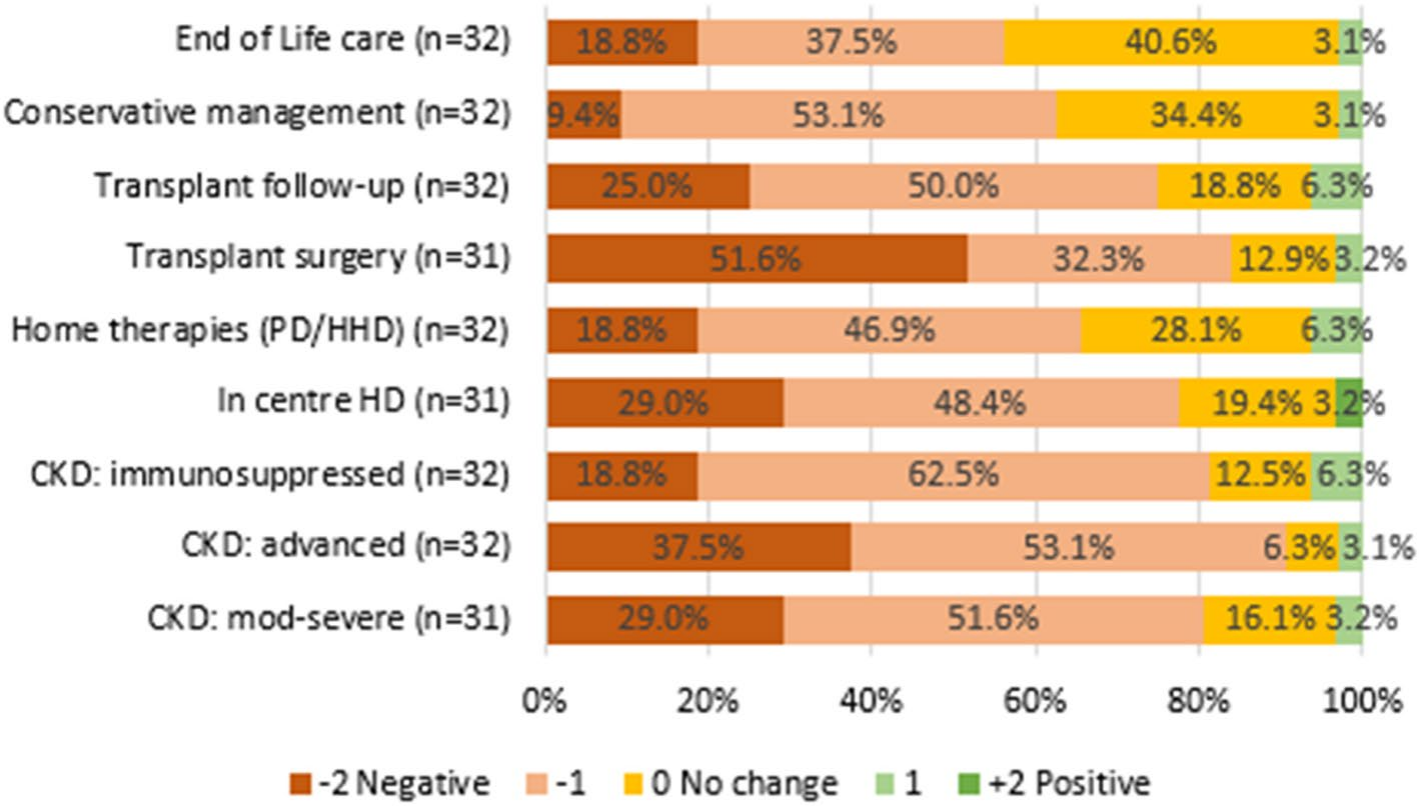
Impact of COVID-19 on services in relation to treatment modality

Centres were asked to estimate the impact of COVID-19 on services according to treatment modality (Fig. 3). Overall impact was overwhelmingly negative. Services for those with advanced CKD, especially those needing to prepare for KRT, those on immunosuppression, and those awaiting transplant surgery were rated as having been most impacted. Those receiving conservative management were thought to have been least affected, though comments suggested those needing end-of-life care were greatly disadvantaged. There were many comments detailing the problems of particular groups. In contrast a handful of centres found positive impacts in relation to each of these services. Centres were asked to outline the main factors constraining service delivery (Table 1). The major issues identified were lack of nurses and lack of beds – including isolation and critical care facilities—but by no means confined to these. Timeliness of information, decision-making and communication were also prominent, as was lack of equipment, especially PPE.

**Table 1:** Impact of issues constraining service delivery during COVID-19

	**1 (Minimal)**	**2**	**3**	**4**	**5 (Significant)**	**Don’t Know**	**N/A**	**Missing**
Staff availability								
Doctors	4 (12.9%)	3 (9.7%)	5 (16.1%)	10 (32.3%)	9 (29.0%)	0	0	17
Nurses	-	-	3 (9.7%)	8 (25.8%)	20 (64.5%)	0	0	17
Other MDT	2 (6.5%)	2 (6.5%)	7 (22.6%)	14 (45.2%)	6 (19.4%)	0	0	17
Admin	3 (10.3%)	4 (13.8%)	9 (31.0%)	7 (24.1%)	6 (20.7%)	1	0	18
Tech	5 (18.5%)	6 (22.2%)	8 (29.6%)	5 (18.5%)	3 (11.1%)	2	0	19
Equipment availability								
PPE	11 (35.5%)	5 (16.1%)	4 (12.9%)	5 (16.1%)	6 (19.4%)	0	0	17
Dialysis machines	20 (64.5%)	3 (9.7%)	3 (9.7%)	4 (12.9%)	1 (3.2%)	0	0	17
HDF fluid	13 (41.9%)	6 (19.4%)	1 (3.2%)	6 (19.4%)	5 (16.1%)	0	0	17
Ventilators	14 (60.9%)	4 (17.4%)	2 (8.7%)	2 (8.7%)	1 (4.3%)	3	4	18
Other	2 (66.7%)	-	-	-	1 (33.3%)	0	3	7
Bed availability								
Isolation	2 (6.5%)	1 (3.2%)	3 (9.7%)	8 (25.8%)	17 (54.8%)	0	0	17
Critical care	1 (3.4%)	4 (13.8%)	3 (10.3%)	8 (27.6%)	13 (44.8%)	0	1	18
Other	1 (33.3%)	-	-	-	2 (66.7%)	0	3	7
Decision making								
Info Timeliness	1 (3.3%)	6 (20.0%)	14 (46.7%)	2 (6.7%)	7 (23.3%)	1	0	17
Communication	1 (3.3%)	6 (20.0%)	13 (43.3%)	4 (13.3%)	6 (20.0%)	1	0	17
Day to day command	2 (6.5%)	12 (38.7%)	11 (35.5%)	2 (6.5%)	4 (12.9%)	0	0	17
COVID-19 command	1 (3.2%)	12 (38.7%)	10 (32.3%)	3 (9.7%)	5 (16.1%)	0	0	17
Other	-	1 (50.0%)	-	-	1 (50.0%)	0	3	7

### Clinical roles

Survey returns concerning medical roles were over 80%. In contrast only 25% provided information on nursing teams. The proportion of centres with medical post vacancies was high, 44% declared more than one vacancy at Specialist Registrar level and 29% at consultant level. Significant changes to medical roles during the pandemic were reported by the majority – mainly rota changes, with new involvement in acute medical intakes and in supporting critical care units. This took place first mainly in wave 2 (September 2020 to June 2021). Nursing vacancies were also high – 50% reporting more than 10. Multiple role changes were reported—specialist nurse deployed to support acute dialysis in critical care, and ward nurses upskilled to support dialysis. New dialysis staff were deployed much earlier than pre-pandemic. Most changes took place first in wave 1. Later nurses played major roles in vaccination roll-out.

Staff absences due to COVID-19 were also high. Around 50% reported that 70–100% of medical staff had been absent for this reason at some point during waves 1 and 2. Returns were less for other staff, but absence rates seemed even higher. Over 80% of centres reported that over 75% of nurses and HCAs underwent absences. Shielding—individuals at higher risk working from home to minimise exposure to infection—caused less disruption. Only 4% of units reported long-term shielding of medical staff. The proportion for nurses and HCAs was a little higher.

There were major differences between centres with respect to the pre-pandemic availability of other professional roles (Table 2). Almost all had access to a dietician and pharmacist embedded in the kidney unit. Fewer had such access to a social worker (48%), psychologist (46%), counsellor (28%) and physiotherapist (25%). Some reported no access to these professionals (27%, 32%, 55% and 14% respectively). Vacancies were also high – over 40% for dietitians, psychologists, and social workers. Absences were mainly due to contracting COVID-19 infection, but redeployment, and shielding, added to service disruptions.

**Table 2:** Availability of multi-disciplinary team prior to COVID-19 pandemic

**Pre-COVID-19 access**	**Kidney service**	**General hospital**	**No access**	**Missing**
Physiotherapist	11 (25.0%)	28 (75.7%)	5 (13.5%)	4
Dietician	44 (100.0%)	-	-	4
Pharmacist	41 (93.2%)	2 (4.5%)	1 (2.3%)	4
Psychologist	20 (45.5%)	10 (22.7%)	14 (31.8%)	4
Psychiatrist	3 (6.8%)	30 (68.2%)	11 (25.0%)	4
Counsellor	10 (22.7%)	10 (22.7%)	24 (54.5%)	4
Social worker	21 (47.7%)	11 (25.0%)	12 (27.3%)	4
Elderly care physician	2 (4.5%)	36 (81.8%)	6 (13.6%)	4
Young adult worker	14 (31.8%)	3 (6.8%)	27 (61.4%)	4
Advocacy officer	5 (11.4%)	9 (20.5%)	30 (68.2%)	4
Palliative care specialist	9 (20.5%)	34 (77.3%)	1 (2.3%)	4
Other	4 (9.1%)	2 (4.5%)	2 (4.5%)	-

### Communication of service changes

Most centres (97%) informed patients how to contact them in case of COVID-19 related concerns or treatment queries. In almost all cases this was not implemented till wave 2.

#### Timing and means of communication (Table 3)

**Table 3:** Communicating COVID-19 related service change information by treatment type

**Informing changes**	**Informed before changes**	**Informed after changes**	**Varied patient to patient**	**Not informed**	**Missing**
Inpatient nephrology	6 (16.2%)	16 (43.2%)	9 (24.3%)	6 (16.2%)	10
Transition	7 (25.9%)	8 (29.6%)	9 (33.3%)	3 (11.1%)	7
Mild/moderate CKD	9 (27.3%)	13 (39.4%)	9 (27.3%)	2 (6.1%)	5
Advanced/low clearance (3b-5)	9 (23.1%)	19 (48.7%)	10 (25.6%)	1 (2.6%)	8
Immunosuppressed	10 (25.6%)	15 (38.5%)	13 (33.3%)	1 (2.6%)	8
In-Centre HD	22 (56.4%)	14 (35.9%)	2 (5.1%)	1 (2.6%)	8
In-Satellite HD	20 (57.1%)	13 (37.1%)	1 (2.9%)	1 (2.9%)	7
HDD/PD	18 (46.2%)	17 (43.6%)	3 (7.7%)	1 (2.6%)	8
Assisted PD	19 (52.8%)	14 (38.9%)	1 (2.8%)	2 (5.6%)	7
Transplant surgery	7 (50%)	7 (50%)	0 (0%)	0 (0%)	3
Transplant Follow-up	16 (41%)	16 (41%)	7 (17.9%)	0 (0%)	7
Living donation	12 (35.3%)	14 (41.2%)	7 (20.6%)	1 (2.9%)	8
Conservative Management	6 (15.8%)	13 (34.2%)	15 (39.5%)	4 (10.5%)	9
End of Life care	5 (14.3%)	10 (28.6%)	16 (45.7%)	4 (11.4%)	7

There was variation between modality groups in relation to communication of service changes. Those on KRT were most often (around 50%) informed before implementation, whereas those attending outpatient nephrology clinics and those receiving conservative management or end of life care, were rarely informed prospectively (< 20%). Delayed communication was attributed to rapid changes in both the evolution of the pandemic and planning guidance. Lack of resources and lack of precise patient data also featured. For most patients (80–100%), especially those considered at high risk – those on dialysis, those with advanced kidney disease, transplanted patients and those on immunosuppression—advice about both shielding and vaccination was delivered individually, mainly by letter. For other groups it was commented that advice was not different to that available to the general population. Suggestions to improve communication included clearer and more timely guidance from government bodies, availability of central sources of patient information and improved IT links to access it, better coordination between primary and secondary care to simplify communication and avoid duplication, better use of social media, and improved patient engagement in planning.

#### Specific provision for vulnerable/underrepresented groups

Few centres offered any specific provision and adjustments for vulnerable and underrepresented groups, including the frail elderly (26%), people with language and communication needs (difficulty with spoken or written English) (24%), young adults (< 30 years) (21%), minority ethnic groups (language and cultural issues) (19%), those living alone (16%), and those with mental health difficulties (11%). A little more support was offered for bereaved or worried carers (28%). Such help as was available was focused on provision of more regular contact by means more appropriate to the individual’s situation.

#### Patient involvement in planning of COVID-19 related service changes

Only a minority of centres involved patients in planning; 28% in both waves, 5% in either wave 1 or 2. Involvement was normally through the presence of a patient representative at meetings of decision-making groups, though in a few instances there were attempts to engage with whole groups of patients. The main reason cited for lack of involvement was the rapidity of change. In fact, the comment was made that there was little opportunity even to consult with staff, and that a ‘command and control’ approach had been necessary.

### Lessons to take forward

**Fig. 4:**
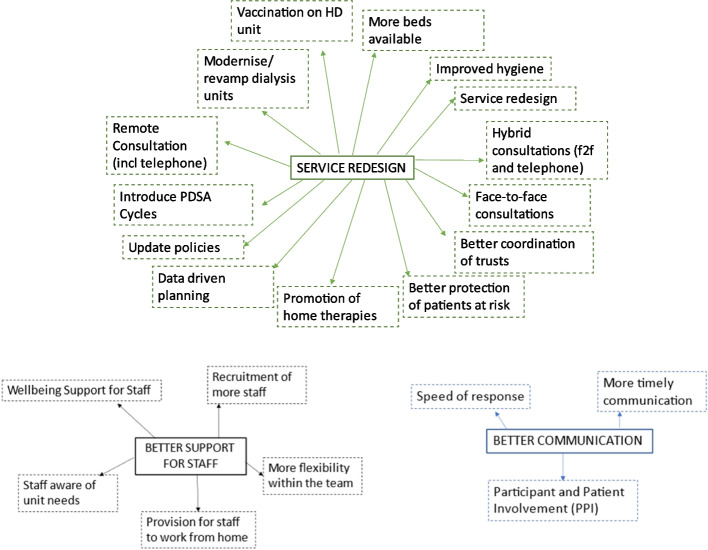
A thematic map to show the themes and codes identified in response to the main lessons from the pandemic that influenced service delivery

Centres were asked to provide three main lessons from the pandemic that would influence their future service delivery. Content analysis of comments generated three themes (Fig. 4). The first, *service redesign* encompassed the need to modernise kidney units with good infection control at the core *“Infrastructure that is modernised and suitable for infectious diseases”;* to improve staffing ratios *“Investment in robust workforce, especially in nursing and KRT workforce”;* and the flexible use of staff *“Importance of working within a connected environment, within Trust, regionally and nationally”*; to improve bed availability *“provision of inpatient bedside dialysis”;* to promote home therapies *“Promotion of home-based therapies”;* and to use telemedicine appropriately *“Many patients like and can be managed virtually”*. The second theme, *better communication* emphasised the need to improve the speed of response, *“Timeliness of communication even when uncertainty”*. The third theme was *better support for staff* with particular emphasis on the need for measures to maintain staff wellbeing, *“Need workforce resilience at all levels, and to look after our staff better”*.

## Discussion

The objective of this national survey of UK kidney centres was to understand the nature, range and degree of variation in kidney care during the COVID-19 pandemic. Responses were obtained from up to 71% of centres. However the data provided about different staff groups varied considerably, being over 80% for medical staff and only 25% for nursing staff. The method used to circulate the questionnaire may have contributed to this, together with the need for multiple responders, and questionnaire length, embodying information requirements for two studies. This was expedient and aimed at reducing responder burden during the pandemic.

We found major changes in kidney services in response to the pandemic. Adaptations were system-wide, affecting all aspects of service provision. Changes were, out of necessity, implemented rapidly usually within the first wave. There was also significant variation in the patterns of adaptation described for different treatment modality groups and between centres.

Service changes occurred for all modality groups though their impact varied. Services for patients with advanced kidney disease and transplant surgical services were judged, in centre responses, to have been the most severely impacted. Patients with advanced kidney disease face critical decisions about KRT modality in preparation for dialysis and/or transplantation or conservative care. Much of this activity was disrupted, with reduction or suspension of vascular access and transplant surgery, and reduced access to specialist nurses. Indeed UK Renal Registry data demonstrate significant reductions between 2019 and 2020 in the proportions of patients starting HD with definitive access and the proportion starting KRT with a transplant [36]. Reduction in face-to-face outpatient activity may also have disproportionately affected these patients given the uncertainties facing them. Less has been written about this group in relation to the pandemic though adaptations to outpatient consultations, access surgery, a relative switch from HD to PD in incident patients have been described [34, 37, 38] as have changes to transplant surgical services [20–24, 39]. Patients undergoing transplant follow-up also faced reduced outpatient opportunities, a switch to virtual consultations and less access to specialist nurses. Changes to in-centre and satellite HD services were considerable, as reported elsewhere [25–29] but the impact on patient experience may have been moderated by some improvements, for instance, by individualisation of transport practices. Home therapies were less impacted.

In comparing centre responses, some adaptations were almost universal, such as those involving outpatient consultations and waiting/arrival/departing procedures for patients receiving HD. However there was wide variation across centres in the frequency of other adaptations. Changes ranged from very frequent, such as PPE use for patients, ‘cohorting’ for patients receiving HD and reduced outpatient activity, to occasional, such as complete suspension of outpatient activity (reported by three centres), reduction in HD duration for all patients (two centres), and delayed dialysis initiation (eight centres). Some changes “bucked the trend”, particularly in relation to PD provision. Many centres reduced PD access provision (13 centres) but in seven this was increased. Many centres reduced PD training, but five increased it. Reconfigurations of support services such as nurse-led clinics, pharmacy and phlebotomy services, involved similar proportions increasing and decreasing provision. In addition, only a small proportion of centres reported making special provision for vulnerable and disadvantaged patients and for worried and bereaved family and carers. This is an important consideration since health inequality is widely documented in kidney care. The impact of the pandemic was experienced differently in relation to social determinants of health [40]. Future planning needs to consider how to advance effective adjustments to safeguard the well-being of those carrying the greatest burden of health inequity.

Drivers of centre variation could include local differences in pandemic severity, local infrastructure and governance, pre-COVID-19 staffing levels, decision-making and communication arrangements, and practice patterns. From the survey we know little about most of these. We do know that there were major staffing difficulties with nursing being particularly hard hit [41], that there was great variation in multidisciplinary team establishment across centres, and that across all staff groups vacancies levels were high. From the staffing perspective at least, the service was ill-prepared for the pandemic. Examining these variations through the lens of centre characteristics could provide other valuable insights. Centre level factors have been shown to be dominant determinants of many outcomes including patients experience [42].

Poor communication of service changes was attributed to the rapid progression of the pandemic, changing planning guidance to centres, and lack of resources. There was little involvement of patients in decision making. Centres viewed the need for service redesign, modernising kidney units to facilitate with good infection control, improving staffing and bed availability, promoting home therapies and optimising use of telemedicine, together with the need for better communication and better support for staff well-being as major lessons for the future.

The study has a number of limitations. It was carried out some time after the onset of the pandemic. This may have influenced recall reliability though may also widened perspectives since a focus of questionnaire was reflection on causes, consequences and learning points. The response rate overall was reasonable in the context of the ongoing pandemic. Responses relating to non-medical staff groups were much lower but overall we think it is likely that these survey findings provide a fair reflection of the response of UK kidney service to the pandemic.

## Conclusion

The response of kidney centres to the pandemic involved adaptations across the whole service. Transplant surgery and services for patients with advanced CKD approaching end-stage kidney disease were particularly impacted. Though there were significant similarities in response there was also wide variation in many responses both at centre level and between modalities. Staffing shortages mainly due to COVID-19 infection and redeployment were compounded by deficiencies in staffing establishments and high vacancy levels. These factors may account for some of the variation between centre responses, but many other issues may be involved. These include type/size of centres, staffing/rota patterns as well as centre culture. Further work is required to carry this learning forward and ensure our patients continue to have access the treatments they need during future severe disruptions including pandemics, natural disasters, and wars. This work should include development of national action plans to facilitate rapid and effective responses to such events.

### Supplementary Information

**Additional file 1.** Copy of survey.

## Data Availability

The datasets used and/or analysed during the current study are available from the corresponding author on reasonable request.
