# Supplementary material for: Impact of the COVID-19 pandemic on services for patients with chronic kidney disease: findings of a national survey of UK kidney centres
Source: BMC Nephrol. 2023 Dec 4;24:356. doi: 10.1186/s12882-023-03344-6 (PMC10696738; doi:10.1186/s12882-023-03344-6)
Supplement: Supplementary file 1 — Additional file 1. Copy of survey. [file 12882_2023_3344_MOESM1_ESM.docx]

## Additional file 1 – Copy of survey


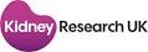

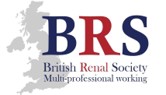

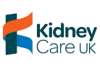


| Mapping the provision of kidney care and associated psychosocial support before and during COVID-19 | Resize font:  \| |
| --- | --- |

We emailed you last week to invite you to take part in this research study. Before you decide whether to participate, it is important that you understand the purpose of data collection and what your involvement will entail.

## **Background**

Professor Ken Farrington (East and North Hertfordshire NHS Trust) and Dr Joe Chilcot (Kings College London) are leading two research studies which review the characteristics of kidney treatment centres and services in the UK, including psychological and social work provision, usually, and during the pandemic:

BRS/KCUK ‘Centre characteristics, practice patterns and the experience of kidney patients during the COVID-19 pandemic’

- To identify characteristics of kidney centres, and the adaptations undertaken in response to the pandemic, which best relate to patient experience of kidney care during Covid-19.

KRUK ‘*A national study of practice patterns in renal services in the identification and management of depression in people with chronic kidney disease.’*

- To better understand how adults with CKD and depression are identified and treated, and to use this information to design effective care pathways.

The two studies have similar enough data requirements in the initial phases that just one survey has been designed and distributed. This ‘two-in-one’ approach is intended to minimise burden on already stretched services.

This survey is being sent to all kidney treatment centres in the UK; Clinical Directors are asked to decide which members of the MDT are best placed to complete each section.

## **Governance**

- This study has been reviewed and approved by The University of Hertfordshire (UH) Health, Science, Engineering and Technology Ethics Committee with Delegated Authority. **The UH protocol number is LMS/SF/UH/04663.**
- The University’s regulation, UPR RE01, 'Studies Involving the Use of Human Participants' can be accessed via this link: <https://www.herts.ac.uk/about-us/governance/university-policies-and-regulations-uprs/uprs> (under letter ‘S’)
- Study data is collected, stored and processed in accordance with GDPR guidelines and UH Standard Operating Procedures (SOP’s). Data will be collected and stored electronically, in a password-protected environment for 24 months, after which time it will be destroyed under secure conditions.
- The Data will be jointly owned by both study teams, analysed as appropriate to meet the aims of each study.

If you have any queries, please contact [kidneyresearch@herts.ac.uk](mailto:kidneyresearch@herts.ac.uk).

Thank you.

## **Survey Instructions**

**Completion of this survey is to be led by the Clinical Director, with support from nursing and other MDT colleagues if you wish.**

**To delegate, please enter their email addresses when prompted.**

**There are further, detailed professional questions, for Psychologists, Counsellors, Psychotherapists and / or Social workers. If you have these in post, you will be asked for their email addresses so that an automatic link can be sent to them.**

**Glossary and timeline**

‘Renal service’ and ‘Renal Centre’ covers all your locations, including satellite.

‘Inpatient’ – receiving care in nephrology ward

‘Outpatient’ – all others, including those on in-centre dialysis treatments.

Please only include staff and equipment that you are directly responsible for, not those which are managed by a private provider

Pandemic waves (UKKW)

Wave One = March to August 2020

Wave Two = September 2020 to June 2021

Lockdown (Institute for Government)

One – 26^th^ March 2020; restrictions eased from 1^st^ June

Two – 5^th^ November 2020; restrictions eased from 2^nd^ December into a 3-4 tier system of restrictions

Three – 6^th^ January 2021; restrictions eased from 8^th^ March

Shielding

21^st^ March 2020 - GP’s asked to identify ‘most clinically vulnerable groups’

1^st^ August 2020 – Shielding paused

13^th^ October to 4^th^ November 2020 – revised guidance issued

Vaccinations

First offered to Priority Groups 1-4 (including over 70’s and the clinically extremely vulnerable): 8^th^ December 2020 – 15^th^ February 2021.

**ASK ALL:**

1. : Name: [FREE TEXT]
2. : Job Title: [FREE TEXT]
3. : Name of Centre: [FREE TEXT]
4. : Which modality patient groups does your renal service care for? (TATA)

| 1. Inpatient nephrology |  |
| --- | --- |
| 1. Transition (young to adult clinics) |  |
| 1. Mild to moderate CKD |  |
| 1. Advanced kidney care / low clearance clinic (Stage 3b-5) |  |
| 1. Immunosuppressed individuals (GN, vasculitis etc) |  |
| 1. In centre haemodialysis |  |
| 1. Satellite haemodialysis |  |
| 1. Home dialysis (HD and PD) |  |
| 1. Assisted PD |  |
| 1. Acute Transplant surgery |  |
| 1. Transplant follow up |  |
| 1. ~~L~~iving donation work up |  |
| 1. Conservative / Supportive care |  |
| 1. End of life care |  |
|  |  |

# Section 1: Renal Service provision during Covid

These questions cover the ***management of inpatient (nephrology) and outpatient) services during Covid, and how Covid impacts were communicated.***

*This section takes up to 30 minutes to complete. You can save and return at any time.*

Renal Service Provisions

ASK All

1. : How were INPATIENT renal services adapted and managed during the COVID crisis? (TATA)

| 1. Cohorting of inpatients by Covid status (green/amber/black) |  |
| --- | --- |
| 1. Isolation ward for renal COVID patients at main centre |  |
| 1. Expansion of dialysis service to ICU to dialyse Covid patients |  |
| 1. Other |  |

ASK if G5 = 4

G5b: How else were INPATIENT renal services adapted and managed during the COVID crisis? (FREE TEXT)

|  |
| --- |

- 1. : Were these service adaptations FIRST implemented during Wave One or Wave Two? (TOO)

|  | Wave One (Mar-Aug 2020) | Wave Two (Sep 2020–Jun 2021) |
| --- | --- | --- |
| Redcap to pull through indicated list from G5 [G5] = “1” |  |  |
| [G5] = “2” etc |  |  |

**Branched question based on the indicated services of G4**

**IF [G4] = ‘6’ AND/OR ‘7’**

1. : How were DIALYSIS PROVISION for IN SATELLITE AND IN CENTRE HAEMODIALYSIS patients adapted and managed during the COVID crisis? (TATA)

| 1. Cohorting Covid-19 positive HD patients |  |
| --- | --- |
| 1. Isolation Covid-19 HD unit at main centre |  |
| 1. Isolation Covid-19 HD at satellite units |  |
| 1. Reduction in HD frequency of dialysis for some patients |  |
| 1. Reduction in HD frequency of dialysis for all patients |  |
| 1. Reduction in HD session length for some patients |  |
| 1. Reduction in HD session length for all patients |  |
| 1. Reduced adequacy testing |  |
| 1. Reduced access salvage procedures |  |
| 1. Provision of PPE for patients |  |
| 1. Changes to who is allowed to accompany patients to ICHD |  |
| 1. Changes to the waiting area / arrival and departure regimes |  |
| 1. Changes to transport |  |
| 1. Changes to rules on food and drink |  |
| 1. Other |  |

**ASK if G6 = 15:**

G6b: How else were renal services for in centre and in satellite patients adapted and managed during the COVID crisis? (FREE TEXT)

|  |
| --- |

- 1. : Were these service adaptations FIRST implemented during Wave One or Wave Two? (TOO)

|  | Wave One (Mar-Aug 2020) | Wave Two (Sep 2020-Jun 2021) |
| --- | --- | --- |
| Redcap to pull through indicated list from G6 [G6] = “1” |  |  |
| [G6] = “2” etc |  |  |

ASK ALL

1. : How were OUTPATIENT renal services (including outpatient clinic activity for dialysis patients) adapted and managed during the COVID crisis? (TATA)

| 1. Remote consultations |  |
| --- | --- |
| 1. Suspension of all outpatient activity |  |
| 1. Reduced outpatient activity |  |
| 1. Full outpatient service |  |
| 1. Changes to nurse led services |  |
| 1. Changes to pharmacy services |  |
| 1. Changes to phlebotomy service |  |
| 1. Changes to transport services |  |
| 1. Other |  |

**If G7 = 3 or 4 ASK**

**G7.1:** How were **Reduced outpatient activity and/or Full outpatient services** conducted: (**TOO**)

|  | mainly face to face | mainly remote consultations | combination |
| --- | --- | --- | --- |
| Reduced outpatient activity |  |  |  |
| Full outpatient services |  |  |  |

**If G7 = 1 or G7.1 remote consultations ticked = 1 ASK**

**G7.2:** How were **remote consultations** conducted **(TATA)**

| Via Telephone |  |
| --- | --- |
| Via Video link |  |

**If Video link is ticked ASK**

**G7.2b:** Please indicate which services were used for **video consultations** i.e Zoom, Teams, Skype etc. **(FREE TEXT)**

|  |
| --- |

**WHERE G7 5-8, ASK**

**G7.3:** How were support services impacted or adapted? **TATA**

|  | **increase** | **decrease** | **reconfiguration** |
| --- | --- | --- | --- |
| **Nurse led services** |  |  |  |
| **Pharmacy services** |  |  |  |
| **Phlebotomy service** |  |  |  |
| **Transport services** |  |  |  |

**If G7 = 9 ASK**

G7b: How else were OUTPATIENT renal services adapted and managed during the COVID crisis? (FREE TEXT)

|  |
| --- |

- 1. : Were these service adaptations FIRST implemented during Wave One or Wave Two? (TOO)

|  | Wave One (Mar-Aug 2020) | Wave Two (Sep 2020-Jun 2021) |
| --- | --- | --- |
| Redcap to pull through indicated list from G7 [G7] = “1” |  |  |
| [G7] = “2” etc |  |  |

**Branched question based on the indicated services of G4**

**IF [G4] = ‘4’ (AKC/LCC)**

1. : How were renal services for patients attending ADVANCED KIDNEY CARE / LOW CLEARANCE CLINICS adapted and managed during the COVID crisis? (TATA)

| 1. Reduced access to specialist nurses |  |
| --- | --- |
| 1. Reduction in HD access surgery |  |
| 1. Suspension of HD access surgery |  |
| 1. Reduced PD access procedures |  |
| 1. Increased PD access procedures |  |
| 1. Delayed dialysis initiation for new starters |  |
| 1. Other |  |

**ASK if G8 = 7**

G8b: How else were renal services for patients ATTENDING ADVANCED KIDNEY CARE / LOW CLEARANCE CLINICS adapted and managed during the COVID crisis? (FREE TEXT)

|  |
| --- |

- 1. : Were these service adaptations FIRST implemented during Wave One or Wave Two? (TOO)

|  | Wave One (Mar-Aug 2020) | Wave Two (Sep 2020-Jun 2021) |
| --- | --- | --- |
| Redcap to pull through indicated list from G8 [G8] = “1” |  |  |
| [G8] = “2” etc |  |  |

**Branched question based on the indicated services of G4**

**IF [G4] = ‘5’ (IMMUNOSUPPRESSED)**

1. : How were renal services for IMMUNOSUPPRESSED INDIVIDUALS adapted and managed during the COVID crisis? (TATA)

| 1. Reduced access to specialist nurses |  |
| --- | --- |
| 1. Reduced access to intensive immunosuppression eg IV cyclophosphamide, rituximab |  |
| 1. Other |  |

**ASK if G9 = 3**

G9b: How else were renal services for IMMUNOSUPPRESSED INDIVIDUALS adapted and managed during the COVID crisis? (FREE TEXT)

|  |
| --- |

G9.1 : Were these service adaptations FIRST implemented during Wave One or Wave Two? (TOO)

|  | Wave One (Mar-Aug 2020) | Wave Two (Sep 2020-Jun 2021) |
| --- | --- | --- |
| Redcap to pull through indicated list from G9 [G9] = “1” |  |  |
| [G9] = “2” etc |  |  |

**Branched question based on the indicated services of G4**

**IF [G4] = ‘8’ AND/OR ‘9’**

1. : How were renal services for HOME DIALYSIS (HD, PD AND Assisted PD) patients adapted and managed during the COVID crisis? (TATA)

| 1. Reduced patient access to specialist nurses |  |
| --- | --- |
| 1. Reduced adequacy testing |  |
| 1. Reduced access salvage procedures |  |
| 1. Reduced PD/HD training |  |
| 1. Enhanced PD/HD training |  |
| 1. Other |  |

**ASK if G10 = 6**

G10b: How else were renal services for HOME DIALYSIS (HD, PD AND Assisted PD) patients adapted and managed during the COVID crisis? (FREE TEXT)

|  |
| --- |

- 1. : Were these service adaptations FIRST implemented during Wave One or Wave Two? (TOO)

|  | Wave One (Mar-Aug 2020) | Wave Two (Sep 2020-Jun 2021) |
| --- | --- | --- |
| Redcap to pull through indicated list from G10 [G10] = “1” |  |  |
| [G10] = “2” etc |  |  |

**Branched question based on the indicated services of G4**

**IF [G4] = ‘10’ AND/OR ‘11’**

1. : How were renal services for TRANSPLANT patients adapted and managed during the COVID crisis? (TATA)

| 1. Reduction of acute transplant surgery |  |
| --- | --- |
| 1. Suspension of acute transplant surgery |  |
| 1. Reduced patient access to specialist nurses |  |
| 1. Other |  |

**ASK if G11 = 4:**

G11b: How else were renal services for TRANSPLANT patients adapted and managed during the COVID crisis? (FREE TEXT)

|  |
| --- |

- 1. : Were these service adaptations FIRST implemented during Wave One or Wave Two? (TOO)

|  | Wave One (Mar-Aug 2020) | Wave Two (Sep 2020-Jun 2021) |
| --- | --- | --- |
| Redcap to pull through indicated list from G11 [G11] = “1” |  |  |
| [G11] = “2” etc |  |  |

**ASK ALL**

1. : Did you make any Covid related service provisions, or offer specific Covid related advice, for any of the following particularly vulnerable patient groups? Please provide brief detail of what was done for each group, and indicate whether this was FIRST provided during Wave One or Wave Two.

|  | No specific provisions | Wave One (Mar-Aug 2020) | Wave Two  (Sep 2020-Jun 2021) | please provide details |
| --- | --- | --- | --- | --- |
| Patients who have difficulty with spoken and or written English |  |  |  | [FREE TEXT] |
| Minority ethnic patient groups |  |  |  | [FREE TEXT] |
| Frail and/or elderly patient groups |  |  |  | [FREE TEXT] |
| Younger patients under 30, including at transition |  |  |  | [FREE TEXT] |
| Bereaved or worried carers |  |  |  | [FREE TEXT] |
| Those who live alone |  |  |  | [FREE TEXT] |
| Those with serious mental health illness |  |  |  | [FREE TEXT] |

**ASK ALL:**

**Patient involvement in planning of Covid related service changes**

1. : Were patients involved in the planning of Covid related service changes, advice or adaptations?
2. Yes: both waves
3. Yes: wave 1 only (Mar-Aug 2020)
4. Yes: wave 2 only (Sep 2020-Jun 2021)
5. No
6. Don’t know

**If G13/1-3 (YES) – go to G13.1 then G13.2**

**If NO / Don’t know – go to G13.2**

- 1. : In what way were patients involved in the planning of Covid related service changes, advice or adaptations? [FREE TEXT]

|  |
| --- |

**ASK ALL**

- 1. : What were the barriers to involving patients in planning of Covid related service changes, advice or adaptations? [FREE TEXT]

|  |
| --- |

**Patient communication**

1. : Were patients informed how to contact the renal centre in case of Covid related concerns or treatment queries?

1. Yes – first in Wave One

2. Yes – first in Wave Two

3. Yes – since July 2021

0. No

**If G14 = 1, 2 or 3 ASK**

- 1. : What were the mechanisms for patients contacting the Centre with Covid related concerns or treatment queries?

| Contact their renal team in the usual way |  |
| --- | --- |
| Other |  |

**If G14.1 = 2**

**G14.1b:** Please specify **other** mechanisms **(FREE TEXT)**

|  |
| --- |

**Service changes**

1. : Were patients made aware by the renal team of Covid related changes to services (TOO)

|  | Before changes were implemented | After changes were implemented | Varied patient to patient | No |
| --- | --- | --- | --- | --- |
| Redcap to pull through indicated list from G4 [G4] = “1” |  |  |  |  |
| [G4] = “2” etc |  |  |  |  |
| None |  |  |  |  |

**WHERE SELECTED AT G15 (apart from NO), SKIP TO G17**

**WHERE NO, GO TO G16**

1. : Have the following patients groups had Covid related changes to services subsequently explained to them?

|  | Yes | No |
| --- | --- | --- |
| Redcap to pull through indicated list from G18 [G18] = “NO” |  |  |

1. : To what extent did the following impact on your ability to communicate Covid related service changes in advance?

|  | 1. No (minimal) Impact | 2 | 3 | 4 | 5. Extreme Impact |
| --- | --- | --- | --- | --- | --- |
| Resource availability (including staff) |  |  |  |  |  |
| Speed of Covid impact |  |  |  |  |  |
| Severity of Covid impact |  |  |  |  |  |
| Time available between planning and implementing changes to services |  |  |  |  |  |
| Changes being interim / evolving over time |  |  |  |  |  |
| Other |  |  |  |  |  |

**If other = 1,2,3,4 or 5 ASK**

**G17b:** Please specify other factors that impacted your ability to communicate **Covid related service changes in advance. (FREE TEXT)**

|  |
| --- |

**Shielding and vaccinations**

1. : To which groups of patients were SHIELDING and VACCINATIONS guidance / advice individually communicated? (TATA)

|  | Shielding | Vaccinations | Neither |
| --- | --- | --- | --- |
| Redcap to pull through indicated list from G4 [G4] = “1” |  |  |  |
| [G4] = “2” etc |  |  |  |

**If neither at any of the above**

- 1. : What prevented you from communicating with those patients on an individual basis? (FREE TEXT)

|  |
| --- |

1. : Generally, across ALL patient groups, which methods of communicating guidance on SHIELDING were used by your centre, and which were most common?

|  | Used at all **(TATA)** | Most common **(TATA)** |
| --- | --- | --- |
| Letter |  |  |
| Phone Call |  |  |
| Text |  |  |
| Email |  |  |
| During Face to Face Consultations |  |  |
| During Online Consultations |  |  |
| Other |  |  |

**If G19 = other**

**G19b: Please specify up to 3 other methods of communication**

G19b1: **Free text**

G19b2: **Free text**

G19b3: **Free text**

- 1. : Was the communication of guidance on **SHIELDING** different for **some patient groups**? Please provide detail below, and indicate which group(s)

G19.1.1: **Free text**

G19.1.2: **Free text**

G19.1.3: **Free text**

**Ask All**

1. : Generally, across **ALL** **patient groups**, which methods of communicating guidance on **VACCINATIONS** were used by your centre, and which were most common?

[Insert option to populate with shielding responses?]

|  | Used at all **(TATA)** | Most common **(TATA)** |
| --- | --- | --- |
| Letter |  |  |
| Phone Call |  |  |
| Text |  |  |
| Email |  |  |
| During Face to Face Consultations |  |  |
| During Online Consultations |  |  |
| Other |  |  |

**If G20 = other**

**G20b: Please specify up to 3 other methods of communication**

G20b1: **Free text**

G20b2: **Free text**

G20b3: **Free text**

- 1. : Was the communication of guidance on **VACCINATIONS** different for **some patient groups**? Please provide detail below, and indicate which group(s)

G20.1.1: **Free text**

G20.1.2: **Free text**

G20.1.3: **Free text**

1. : How, if at all, could communications on **service changes, vaccinations and / or shielding** have been improved? Please specify for which groups, for what communications and how, if appropriate. **[FREE TEXT]**

|  |
| --- |

## **STAFFING**

This section assumes that you have the following staff in your workforce, and all questions are structured accordingly.

| 1. Nephrologist |
| --- |
| 1. General Nurse |
| 1. Specialist Nurse |
| 1. Healthcare Assistant |

You will be given the option to send questions relating to nursing staff provisions to your lead nurse or nominated deputy. Please tick any questions you would like to forward and then enter the email of the nominated person when prompted.

**ASK ALL:**

1. : Prior to COVID-19, which of the following care professionals did patients routinely have access to? Please indicate whether access was part of the renal service, or part of the general hospital/community provision? (tick all that apply)

|  | As part of the renal service | As part of the general hospital / community provision | No routine access, pre-COVID |
| --- | --- | --- | --- |
| 1. Transplant surgeon |  |  |  |
| 1. Physiotherapist |  |  |  |
| 1. Dietitian |  |  |  |
| 1. Pharmacist |  |  |  |
| 1. Psychologist |  |  |  |
| 1. Psychiatrist |  |  |  |
| 1. Counsellor |  |  |  |
| 1. Social worker |  |  |  |
| 1. Elderly care physician |  |  |  |
| 1. Young adult worker |  |  |  |
| 1. Advocacy officer |  |  |  |
| 1. Palliative care specialist |  |  |  |
| 1. ~~Other~~ |  |  |  |

The next few questions are about the structure and Covid impact on the multi-disciplinary team:

- Please provide numbers or proportions where indicated: these can be estimates.
- Staff may fall in to more than one category, so totals do not need to add to 100%
- If you wish to refer any of these short MDT sessions to a colleague, please enter that person’s email address when prompted to do so.

1. : Please provide details about your MEDICAL STAFFING (please complete all that apply)

|  | WTE | Vacancies |
| --- | --- | --- |
| 1. Consultants | [FREE TEXT] | [FREE TEXT] |
| 1. Staff Grade, Specialty and Associate Specialist (SAS) doctors | [FREE TEXT] | [FREE TEXT] |
| 1. Specialist Registrars/ Clinical Fellow (Registrar Grade) | [FREE TEXT] | [FREE TEXT] |
| 1. Research Fellow Registrar Grade | [FREE TEXT] | [FREE TEXT] |
| 1. Junior Middle Grade (CMT1/2, ST1/2, SHO) | [FREE TEXT] | [FREE TEXT] |
| 1. FY 1/2 | [FREE TEXT] | [FREE TEXT] |

1. : How did the role of MEDICAL STAFFING change during the Covid pandemic and when were these first implemented?

|  | Wave One (Mar-Aug 2020) | Wave Two (Sep 2020-Jun 2021) |
| --- | --- | --- |
| 1. Rota changes |  |  |
| 1. Involvement in medical take |  |  |
| 1. ITU involvement |  |  |
| 1. Vaccinations |  |  |
| 1. Other |  |  |

**IF G25 for other is tick for wave 1**

**G24b:** Please specify any **other** changes to **MEDICAL STAFFING** roles during **Wave One** of the Covid pandemic. **(Free Text)**

|  |
| --- |

**IF G25 for other is tick for wave 2**

**G24c:** Please specify any **other** changes to **MEDICAL STAFFING** roles during **Wave Two** of the Covid pandemic. **(Free Text)**

|  |
| --- |

1. : Please give approximate proportions of MEDICAL STAFF effected by the following:

|  | Approximate proportion (%) who experienced absences due to impact of Covid on self / family | Approximate proportion (%) who had to shield due to Covid |
| --- | --- | --- |
| Consultants and SAS | [FREE TEXT] | [FREE TEXT] |
| Doctors in training roles | [FREE TEXT] | [FREE TEXT] |

**ASK ALL**

1. : Please provide details about your NURSING AND HEALTHCARE ASSISTANT staff (please complete all that apply)

|  | WTE | Vacancies |
| --- | --- | --- |
| 1. Qualified Nurses | [FREE TEXT] | [FREE TEXT] |
| 1. Nurses in training | [FREE TEXT] | [FREE TEXT] |
| 1. Qualified Healthcare assistants (HCA’s) | [FREE TEXT] | [FREE TEXT] |
| 1. HCA’s in training | [FREE TEXT] | [FREE TEXT] |

If you would like to send this question to your Lead Nurse or Nominated deputy please tick here

**G27a:** Please specify what changes to **NURSING AND HEALTHCARE ASSISTANT** roles took place during **Wave One** of the Covid pandemic. **(Free Text)**

|  |
| --- |

If you would like to send this question to your Lead Nurse or Nominated deputy please tick here

**IF G28 for other is tick for wave 2**

**G27b:** Please specify what changes to **NURSING AND HEALTHCARE ASSISTANT** roles took place during **Wave Two** of the Covid pandemic. **(Free Text)**

|  |
| --- |

If you would like to send this question to your Lead Nurse or Nominated deputy please tick here

1. : Please give approximate proportions of NURSING AND HEALTHCARE ASSISTANT staff effected by the following:

|  | Approximate proportion (%) who experienced absences due to impact of Covid on self / family | Approximate proportion (%) who had to shield due to Covid | Approximate proportion (%) who were redeployed out of the service | Approximate proportion (%) who were redeployed into the service |
| --- | --- | --- | --- | --- |
| Nurses | [FREE TEXT] | [FREE TEXT] | [FREE TEXT] | [FREE TEXT] |
| HCA’s | [FREE TEXT] | [FREE TEXT] | [FREE TEXT] | [FREE TEXT] |

If you would like to send this question to your Lead Nurse or Nominated deputy please tick here

1. : Please provide details about Other MTD staff (please complete all that apply) Pull through from those picked at G22 in renal unit

|  | WTE | Vacancies |
| --- | --- | --- |
| 1. Dietitians | [FREE TEXT] | [FREE TEXT] |
| 1. Physiotherapists | [FREE TEXT] | [FREE TEXT] |
| 1. Pharmacists | [FREE TEXT] | [FREE TEXT] |
| 1. Technicians | [FREE TEXT] | [FREE TEXT] |
| 1. Administrative Staff | [FREE TEXT] | [FREE TEXT] |
| 1. Other MTD staff in the unit (Other than Psychology and Social work staff) | [FREE TEXT] | [FREE TEXT] |

If you would like to send this question to your Lead Nurse or Nominated deputy please tick here

1. : Please give approximate proportions of Other MTD staff effected by the following:

|  | Approximate proportion (%) who experienced absences due to impact of Covid on self / family | Approximate proportion (%) who had to shield due to Covid | Approximate proportion (%) who were redeployed out of the service | Approximate proportion (%) who were redeployed into the service |
| --- | --- | --- | --- | --- |
| Dietitians | [FREE TEXT] | [FREE TEXT] | [FREE TEXT] | [FREE TEXT] |
| Physiotherapists | [FREE TEXT] | [FREE TEXT] | [FREE TEXT] | [FREE TEXT] |
| Pharmacists | [FREE TEXT] | [FREE TEXT] | [FREE TEXT] | [FREE TEXT] |
| Technicians | [FREE TEXT] | [FREE TEXT] | [FREE TEXT] | [FREE TEXT] |
| Administrative Staff | [FREE TEXT] | [FREE TEXT] | [FREE TEXT] | [FREE TEXT] |
| Other MTD staff in the unit (Other than Psychology and Social work staff) | [FREE TEXT] | [FREE TEXT] | [FREE TEXT] | [FREE TEXT] |

If you would like to send this question to your Lead Nurse or Nominated deputy please tick here

1. : How substantial has the impact been of long Covid (signs and symptoms that develop during or following an infection consistent with COVID-19 which continue for more than 12 weeks, not explained by an alternative diagnosis) on ALL staff? Where 1 = minimal and 5 = substantial

1 2 3 4 5

**CD / LEAD NURSE – ASK ALL**

## **PSYCHOLOGICAL IMPACT OF COVID 19 ON STAFF**

1. : As a service, BEFORE COVID-19, which of the following did you provide with respect to psychosocial care for staff? (tick all that apply)

| Debrief after critical incidents |  |
| --- | --- |
| Schwartz rounds/Team Time |  |
| Provide in-house psychological support / counselling |  |
| Signpost access to external to the unit psychological support / counselling |  |
| Screening for psychological distress / PTSD |  |
| Peer support groups |  |

- 1. : Did the provision of the following methods of psychosocial care for staff increase, stay the same, or reduce since Covid 19?

|  | Increased | Stayed the same | Reduced | Don’t know |
| --- | --- | --- | --- | --- |
| Redcap to pull through indicated list from G32 = “1” |  |  |  |  |
|  |  |  |  |  |

1. : As a service, SINCE COVID-19, which of the following have you begun to provide with respect to psychosocial care for staff? (tick all that apply)

Redcap to pull through those NOT selected at G27

| Debrief after critical incidents |  |
| --- | --- |
| Schwartz rounds/Team Time |  |
| Provide in-house psychological support / counselling |  |
| Signpost access to external to the unit psychological support / counselling |  |
| Screening for psychological distress / PTSD |  |
| Peer support groups |  |
| Other |  |

If G33 = Other

G33b: Please specify Other services that you have begun to provide with respect to psychosocial care for staff

G34b1: FREE TEXT

G34b2: FREE TEXT

G34b3: FREE TEXT

1. : Were these support mechanisms FIRST implemented during Wave One or Wave Two?

Redcap to pull through those selected at G34b

|  | Wave One | Wave Two | Don’t know |
| --- | --- | --- | --- |
| Debrief after critical incidents |  |  |  |
| Schwartz rounds/Team Time |  |  |  |
| Provide in-house psychological support / counselling |  |  |  |
| Signpost access to external to the unit psychological support / counselling |  |  |  |
| Screening for psychological distress / PTSD |  |  |  |
| Peer support groups |  |  |  |
| Pull in the ‘other’ categories G34b1 etc |  |  |  |

## **SERVICE PROVISION DURING COVID**

1. : How much was your unit’s care response to Covid influenced by guidance from the following sources? (TATA) Likert scale

|  | 1 Minimal | 2 | 3 | 4 | 5 Significant |
| --- | --- | --- | --- | --- | --- |
| Government |  |  |  |  |  |
| Renal Association / BRS |  |  |  |  |  |
| Other national (e.g. NICE) |  |  |  |  |  |
| Professional bodies eg Transplant |  |  |  |  |  |
| Regional Renal Network |  |  |  |  |  |
| Trust provision |  |  |  |  |  |
| Bespoke renal unit |  |  |  |  |  |
| Charity |  |  |  |  |  |

## **IMPACT OF COVID 19 ON SERVICES**

1. : Could you estimate the overall impact of Covid-19 on healthcare services for the following patient groups?

0 = no change
-2 = negative impact,
2 = positive impact

|  | -2 Negative Impact | -1 | 0  No change | 1 | 2 Positive Impact |
| --- | --- | --- | --- | --- | --- |
| CKD; Moderate -severe |  |  |  |  |  |
| CKD; Advanced – preparing for renal replacement / supportive care |  |  |  |  |  |
| CKD; Immunosuppressed |  |  |  |  |  |
|  | | | | | |
| **Haemodialysis** Centre/satellite |  |  |  |  |  |
| **Haemodialysis** Home HD, PD including assisted PD |  |  |  |  |  |
|  | | | | | |
| **Transplant –** post-surgery follow up |  |  |  |  |  |
| **Transplant** longer term ollow-up |  |  |  |  |  |
| **Conservative care** |  |  |  |  |  |
| **End of life care** |  |  |  |  |  |

1. : Please can you elaborate, as to which aspect of the service experienced the most impact, whether positive or negative, and the main reasons for this [FREE TEXT]

|  |
| --- |

## **RESOURCE ISSUES**

1. : Overall, to what extent did the following factors impact on service delivery during Covid? [Make banners different colours]

|  | Minimal |  |  |  | Significant | Don’t know | N/A |
| --- | --- | --- | --- | --- | --- | --- | --- |
|  | 1 | 2 | 3 | 4 | 5 |  |  |
| **Staff availability** |  |  |  |  |  |  |  |
| 1. Doctors |  |  |  |  |  |  |  |
| 1. Nurses |  |  |  |  |  |  |  |
| 1. Other MDT members |  |  |  |  |  |  |  |
| 1. Technical staff |  |  |  |  |  |  |  |
| 1. Administrative/support staff |  |  |  |  |  |  |  |
| **Availability of equipment** |  |  |  |  |  |  |  |
| 1. PPE |  |  |  |  |  |  |  |
| 1. Dialysis machines |  |  |  |  |  |  |  |
| 1. Haemofiltration machines / fluid |  |  |  |  |  |  |  |
| 1. Ventilators |  |  |  |  |  |  |  |
| 1. Other |  |  |  |  |  |  |  |
| **Bed availability** |  |  |  |  |  |  |  |
| 1. Isolation facilities |  |  |  |  |  |  |  |
| 1. Critical care capacity |  |  |  |  |  |  |  |
| 1. Other |  |  |  |  |  |  |  |
| **Decision making** |  |  |  |  |  |  |  |
| 1. Timeliness of information |  |  |  |  |  |  |  |
| 1. Clarity of communication |  |  |  |  |  |  |  |
| 1. Command structure (day to day) |  |  |  |  |  |  |  |
| 1. Command structure (Covid specific) |  |  |  |  |  |  |  |
| 1. Other |  |  |  |  |  |  |  |

If G38 factor 10 is ticked

G38.10b: Please specify Other Equipment factor [FREE TEXT]

|  |
| --- |

If G38 factor 13 is ticked

G38.13b: Please specify Other Bed availability factor [FREE TEXT]

|  |
| --- |

If G39 factor 18 is ticked

G38.18b: Please specify Other Decision making factor [FREE TEXT]

|  |
| --- |

1. : What are the main lessons from the pandemic that will influence your service delivery in the future? Please describe up to three in order of significance (1 highest)

G39b1: 1 **[FREE TEXT]**

G39b2: 2 **[FREE TEXT]**

G39b3: 3 **[FREE TEXT]**

**Psychological and social work provision, usually and during Covid**

## **Centre level psychological provision**

*This section collects centre level data, to be completed by all centres, regardless of specialist psychosocial provision. There is an opportunity to send modules on psychologist, counselling and social work provision to those colleagues, in the next section.*

1. : Does your renal service have local pathways for the identification and management of depression and / or anxiety?
2. Yes
3. No
4. Unsure
5. : Does your renal medical staff receive training and education regarding the identification and management of depression and / or anxiety?
6. Yes
7. No
8. Unsure

If G41 = ‘1’

- 1. : Who delivers this typically**?**

[FREE TEXT]

- 1. : Is this delivered in the MDT setting?

1. No
2. Yes
3. : Do you have doctors in your renal team with a special interest in psychological medicine/mental health?
4. Yes
5. No
6. Unsure
7. : Do your renal nursing staff receive training and education regarding the identification and management of depression and / or anxiety?
8. Yes
9. No
10. Unsure

If you would like to send this question to your Lead Nurse or Nominated deputy please tick here

If G43 = ‘1’

- 1. : Who delivers this typically**?**

[FREE TEXT]

If you would like to send this question to your Lead Nurse or Nominated deputy please tick here

- 1. : Is this delivered in the MDT setting?

1. No
2. Yes

If you would like to send this question to your Lead Nurse or Nominated deputy please tick here

1. : Do you have nurses who are mental health champions or those with a special interest in the topic?
2. Yes
3. No
4. Unsure

If you would like to send this question to your Lead Nurse or Nominated deputy please tick here

1. : Are you aware of the NICE guidelines “Depression in adults with a chronic physical health problem: recognition and management” [CG91]? Or National equivalence (e.g. SIGN).
2. No
3. Yes
4. : Who typically prescribes anti-depressants to your patients (tick all that apply)

| 1. Nephrologists |  |
| --- | --- |
| 1. MDT |  |
| 1. Psychiatrist |  |
| 1. GP |  |
| 1. External professionals (e.g. Mental health team) |  |
| 1. No specific policy |  |

1. : Who typically reviews anti-depressants use amongst your patients (tick all that apply):

| 1. Nephrologists |  |
| --- | --- |
| 1. With discussion with the MDT |  |
| 1. Psychiatrist |  |
| 1. GP |  |
| 1. External professionals (e.g. Mental health team) |  |
| 1. No specific policy |  |

1. : Do you have a policy for the withdrawal of anti-depressants?
2. Yes
3. No
4. Unsure

**If G48 = ‘1’**

- 1. : Who manages the withdrawal of anti-depressants?

| 1. Nephrologists |  |
| --- | --- |
| 1. With discussion with the MDT |  |
| 1. Psychiatrist |  |
| 1. GP |  |
| 1. External professionals (e.g. Mental health team) |  |

1. : As an approximation (%), in your service, how many patients in each of the following treatment groups do you think are usually taking anti-depressants

|  | estimate % | Don’t know |
| --- | --- | --- |
| Mild to moderate CKD  Advanced kidney care / low clearance clinic | **[FREE TEXT]** |  |
| In centre / satellite haemodialysis | **[FREE TEXT]** |  |
| Home dialysis (HD, PD and assisted PD) | **[FREE TEXT]** |  |
| Transplant follow up | **[FREE TEXT]** |  |
| Conservative / Supportive care | **[FREE TEXT]** |  |

1. : During COVID did this increase / decrease / stay the same?

|  | Increase | Decrease | Stay the same | Don’t know |
| --- | --- | --- | --- | --- |
| Mild to moderate CKD  Advanced kidney care / low clearance clinic |  |  |  |  |
| In centre / satellite haemodialysis |  |  |  |  |
| Home dialysis (HD, PD and assisted PD) |  |  |  |  |
| Transplanted |  |  |  |  |
| Conservative / Supportive care |  |  |  |  |

## Centre level social work provision

1. : Does your unit staff receive training and education concerning the management of social issues?
2. No
3. Whole Team Training
4. Medical staff only
5. Nursing staff only
6. Other MDT members regarding the identification and management of social issues?

**If G51 = ‘1’, ‘2’, ‘3’ or ‘4’**

- 1. : Is this delivered in the MDT setting?

1. Yes
2. No
3. Don’t know

**If G51.1 = ‘1’**

- 1. : Who delivers social issue training in the MDT setting?

**[FREE TEXT]**

1. : Do you have nurses who are social care champions or those with a special interest in social issues?
2. Yes
3. No
4. Unsure
5. : Are social issues identified on patient referral into kidney care?
6. Yes – specifically investigated (e.g. in a nurse or doctor taking a social history)
7. Yes – as part of referral from another service
8. Yes – if a patient mentions them

0. No

1. don’t know
2. : Are social issues identified in prevalent patients? (i.e., patients already established in the unit)?
3. Yes – specifically investigated (e.g. in a nurse or doctor taking a social history)
4. Yes – as part of referral from another service
5. Yes – if a patient mentions them
6. No
7. don’t know

| **INSTRUCTION 1: In the information provided so far, you have indicated that your service has:**  **~~IF 9, OR 10, OR 11, OR 12 AND 13 at G18:~~**  ~~a)~~ **~~In house psychological support AND social work provision~~**~~. Please enter the name and email of the lead psychologist; counsellor or therapist AND social worker in order for them to receive some questions about their service and care.~~  ~~Psychologist name~~ **~~FREE TEXT~~**  ~~Psychologist email~~ **~~FREE TEXT~~**  ~~Social worker name~~ **~~FREE TEXT~~**  ~~Social worker email~~ **~~FREE TEXT~~**  ~~PSY AND SOCIALWORKER TO RECEIVE THEIR MODULE FROM MOODMAPS FULL~~  **~~RedCap to now Filter TO G57:~~**  **~~IF 9 OR 10, OR 11, OR 12 at G18:~~**  b) **In house psychological support provision**. In the information provided so far, you have indicated that your serviced has:  Psychologist name **free text**  Psychologist email **free text**  PSY TO RECEIVE THEIR MODULE FROM MOODMAPS PSY AND CD TO SEE QUESTIONS ABOUT HOW SOCIAL WORK NEEDS ARE REFERRED OUT /HANDLED  **RedCap to now Filter TO G55:**  **IF 13 at G18:**  c) **In house social work provision**. Please enter the name and email of the lead social worker ___________ in order for them to receive some questions about their service and care. Since you have indicated no in house psychological support provision, please complete the following few questions about referral and management  SOCIAL WORKER TO RECEIVE THEIR MODULE FROM MOODMAPS SOCIAL AND CD TO SEE QUESTIONS ABOUT HOW PSYCHOLOGICAL NEEDS ARE REFERRED OUT /HANDLED – ci below  **RedCap to now Filter TO G54 but filter past G55 to G57:**  **IF NONE OF 9 OR 10 OR 11 OR 12 OR 13 at G18:**  d) **Neither psychological or social work support provision in house**. Please complete the following few questions about referral and management |
| --- |

**WHERE (c) or (d) AT INSTRUCTION 1 (IE, NO PSYCHOLOGICAL PROVISION) ASK:**

1. : If a patient has psychological or mental health issues, how is care typically provided?

(Tick all that apply – both normally and during Covid)

| Source | Normally | During Covid |
| --- | --- | --- |
| 1. GP |  |  |
| 1. Renal service referral to IAPT |  |  |
| 1. Nephrology referral to Mental Health team |  |  |
| 1. Nephrology prescription and management of psychotropic medication(s) |  |  |
| 1. Referral to other MDT provision |  |  |
| 1. Referral to other extra provision |  |  |
| 1. Signpost to KCUK support services |  |  |
| 1. Signpost to other MH support organisation |  |  |
| 1. Signpost to other renal support organisation |  |  |

**IF G56 = ‘5’ OR ‘6’ OR ‘8’ OR ‘9’**

- 1. : Please specify other services

| 1 | [FREE TEXT] |
| --- | --- |
| 2 | [FREE TEXT] |
| 3 | [FREE TEXT] |

**WHERE (b) or (d) AT INSTRUCTION 1 (IE, NO SOCIAL WORK PROVISION) ASK:**

1. : When a patient is identified as having a social issue, do you have a process on how staff should deal with this? Whether formal or informal, please describe below.

**[FREE TEXT]**

1. : What are the normal routes of referral for patients with social issues? Please tick all that apply

|  | Usually | During Covid |
| --- | --- | --- |
| Hospital social work team |  |  |
| Community based adult social work teams |  |  |
| GP |  |  |
| Other |  |  |

**GNR: Review of Questions ticked to go to the lead nurse or nominated deputy**

## CENTRE DETAIL

We would like to ask you a few questions about your perceptions of centres and future research opportunities.

1. : Which centre do you compare yourselves to when national data is published?

[FREE TEXT]

1. : What are the characteristic(s) of that centre that makes you feel it is similar to you?

[FREE TEXT]

1. : If you would like to receive a copy of the study report later in the year, please insert your email address below.

[FREE TEXT]

1. : Would be willing to receive information about further study centre service provision? Your contact details will only be shared with members of the research team.

[FREE TEXT]

Thank you for your time. If you have any queries, please contact [kidneyresearch@herts.ac.uk](mailto:kidneyresearch@herts.ac.uk). We will follow up with centres in due course.

# **Two: Psychological Support Module**

**TO BE ANSWERED BY THE COUNSELLOR / PSYCHOTHERAPIST / PSYCHOLOGIST**

**AS IDENTIFIED IN THE INSTRUCTION SECTION**

1. : Please tell us about the staffing numbers relating to the psychosocial team in which patients have routine access to.

|  | Number of WTE (qualified) | Number of vacancies | Number of WTE staff in training | Approximate number who experienced absences due to impact of Covid on self / family | Approximate number who had to shield due to Covid | Approximate number who were redeployed out of the service | Approximate number who were redeployed into the service |
| --- | --- | --- | --- | --- | --- | --- | --- |
| Psychologist | [FREE TEXT] | [FREE TEXT] | [FREE TEXT] | [FREE TEXT - INTEGER] | [FREE TEXT] | [FREE TEXT] | [FREE TEXT] |
| Psychiatrist | [FREE TEXT] | [FREE TEXT] | [FREE TEXT] | [FREE TEXT] | [FREE TEXT] | [FREE TEXT] | [FREE TEXT] |
| Counsellor | [FREE TEXT] | [FREE TEXT] | [FREE TEXT] | [FREE TEXT] | [FREE TEXT] | [FREE TEXT] | [FREE TEXT] |
| Assistant Psychologists | [FREE TEXT] | [FREE TEXT] | [FREE TEXT] | [FREE TEXT] | [FREE TEXT] | [FREE TEXT] | [FREE TEXT] |
| Social worker | [FREE TEXT] | [FREE TEXT] | [FREE TEXT] | [FREE TEXT] | [FREE TEXT] | [FREE TEXT] | [FREE TEXT] |
| Other | [FREE TEXT] | [FREE TEXT] | [FREE TEXT] | [FREE TEXT] | [FREE TEXT] | [FREE TEXT] | [FREE TEXT] |

1. : As lead of the renal psychological support service, please indicate your profession (tick all that apply):

| 1. Practitioner Psychologist |  |
| --- | --- |
| 1. Counsellor |  |
| 1. Psychiatrist |  |
| 1. Other |  |

**If P2 = 4 ASK**

**P2b:** Please specify the profession **(FREE TEXT)**

|  |
| --- |

1. : Which modality patient groups does your renal psychosocial service offer support to? (tick all that apply)

| 1. Inpatient nephrology |  | | |
| --- | --- | --- | --- |
| 1. Transition (young to adult clinics) |  | | |
| 1. Mild to moderate CKD |  | | |
| 1. Advanced kidney care / low clearance clinic (Stage 3b-5) |  | | |
| 1. Immunosuppressed individuals (GN, vasculitis etc) |  | | |
| 1. In centre haemodialysis |  | | |
| 1. Satellite haemodialysis |  | | |
| 1. Home dialysis (HD and PD) |  | | |
| 1. Assisted PD |  | | |
| 1. Acute Transplant surgery |  | | |
| 1. Transplant follow up |  | | |
| 1. Living donation |  | | |
| 1. Conservative / Supportive care |  | | |
| 1. End of life care | |  |  |

1. : Where do you get your referrals from; please give approximate proportions of how many are in-patients or out-patients?
2. In-patients **[FREE TEXT]%**
3. Out-patients **[FREE TEXT]%**
4. : Which of these psychosocial services do you offer patients? (tick all that apply

| \| 1. Complex psychological assessment, formulation and psychological intervention \| ☐ \| \| --- \| --- \| \| 1. Low intensity psychological intervention \| ☐ \| \| 1. Emergency hotlines (internal / external) \| ☐ \| \| 1. one to one counselling \| ☐ \| \| 1. screening for Psychological distress including depression and anxiety \| ☐ \| \| 1. Cognitive screening and neuropsychological assessment \| ☐ \| \| 1. bereavement counselling \| ☐ \| \| 1. support for carers \| ☐ \| \| 1. signposting to other services \| ☐ \| \| 1. Peer support group services \| ☐ \| \| 1. patient support leaflets \| ☐ \| \| 1. Online support \| ☐ \| \| 1. Other \| ☐ \| |  |
| --- | --- | --- | --- | --- | --- | --- | --- | --- | --- | --- | --- | --- | --- | --- | --- | --- | --- | --- | --- | --- | --- | --- | --- | --- | --- | --- | --- |

**IF P5 10 is ticked**

- 1. : Please specify other services

| 1. **[FREE TEXT]** |
| --- |
| 1. **[FREE TEXT]** |
| 1. **[FREE TEXT]** |

1. : Have any of these psychosocial services for patients increased, stayed the same or reduced since COVID-19? (tick all that apply)

|  | Increased | Stayed the same | Reduced | Don’t know |
| --- | --- | --- | --- | --- |
| Redcap to pull through indicated list from P5 = “1” |  |  |  |  |
|  |  |  |  |  |

1. : From your perspective, what is the most important gap in psychosocial provision in your service for patients and / or carers at the moment?

**[FREE TEXT]**

1. : Is there a formal referral pathway into your service
2. No
3. Yes
4. Both formal and informal
5. : What are the modes of access into your service (for assessment or treatment) (tick all that apply)

|  | Usually | During Covid |
| --- | --- | --- |
| 1. Self-referral |  |  |
| 1. GP |  |  |
| 1. From the medical or surgical team |  |  |
| 1. From the MDT |  |  |
| 1. Via proactive screening for mental health/other difficulties |  |  |
| 1. Family/carer |  |  |
| 1. Via external bodies (for example KCUK) |  |  |
| 1. Social worker |  |  |
| 1. Care navigator |  |  |
| 1. Other |  |  |

**IF p9 = 10**

P9b: Please specify

[FREE TEXT]

1. : What is the typical average number of therapy sessions PER PATIENT your service provides?

| 1-3 sessions |  |
| --- | --- |
| 4-6 sessions |  |
| 7-10 sessions |  |
| 11-15 sessions |  |
| >16 sessions |  |
| Varies considerably |  |
| Don’t know |  |

1. : Is there a maximum number of sessions offered to an individual?

0.No

1.Yes

P11.1 Please specify [FREE TEXT]

1. : Typically, how frequent are the therapy sessions?
2. Weekly
3. Fortnightly
4. Monthly sessions
5. More than 1 month between sessions
6. Don’t know
7. : Approximately how many NEW patients does your service see per month?

**[FREE TEXT]**

1. : Approximately how many FOLLOW-UP patients does your service see per month?

**[FREE TEXT]**

1. : How has caseload changed during COVID?
2. No change
3. Increase in caseloads
4. Decrease in caseloads
5. : How long is the waiting list for new referrals? (tick all that apply)

|  | Usually | During Covid |
| --- | --- | --- |
| Less than 1 month |  |  |
| Between 1-3 months |  |  |
| More than 3 months |  |  |

1. : Do you provide any information / psychoeducation to people on the waiting list?
2. No
3. Yes

**If P17 = ‘1’**

P17b: Please specify

1. : Does your psychosocial support service refer patients to the Improving Access to Psychological Therapies (IAPT) service? (tick all that apply)

|  | Usually | During Covid |
| --- | --- | --- |
| 1. Directly |  |  |
| 1. Indirectly Via GP |  |  |
| 1. Signpost patients to self-referral pathway |  |  |
| 1. Other |  |  |
| 1. No |  |  |

**IF P18 = ‘4’**

**P18b** : Please specify

[FREE TEXT]

P19: Do you proactively screen for depression and/or anxiety in the following modalities

| Redcap to pull through indicated list from P3 [P3] = ‘1’ | No  Yes |
| --- | --- |
| P3 = ‘2’ |  |

**If yes to any from P19**

P19.1: Which screening measures do you use (tick all that apply)

1. Distress thermometer
2. Hospital Anxiety Depression Scale (HADS)
3. Patient Health Questionnaire-2 (PHQ-2)
4. Patient Health Questionnaire-4 (PHQ-4)
5. Patient Health Questionnaire-9 (PHQ-9)
6. Generalised Anxiety Scale-2 (GAD-2)
7. Generalised Anxiety Scale-7 (GAD-7)
8. Patient Health Questionnaire- Anxiety Depression Scale (PHQ-ADS)
9. Other

**P19.1b:** Please Specify **Other** measures

**[FREE TEXT]**

**If yes to any from P19**

P19.2 : How often do you screen?

1. Monthly
2. 6 monthly
3. Annually
4. At key points in the treatment pathway

**IF P19.2 = ‘4’**

**P19.2b:** Please specify the **Key Points** in the pathway

**[FREE TEXT]**

**If yes to any from P19**

P19.3 : Who typically screens?

1. Psychologist/therapist/counsellor
2. Nurse
3. Doctor
4. Other

**IF P19.3 = ‘4’**

**P19.3b:** Please specify **Other** people who typically screen

**[FREE TEXT]**

**If yes to any from P19**

P19.4 : What decisions are made based on the screening results?

1. Referral decisions

2. Treatment decisions

3. Monitoring of symptoms

4. Evaluation of treatment interventions

5. Other

**If p.19.4 = 5**

**P19.5b:** Please specify **Other** decisions

1. : Are you aware of the relevant NICE guidelines: *“Depression in adults with a chronic physical health problem: recognition and management”* [CG91]?
2. No
3. Yes

**If P20 = ‘1’**

- 1. : Does your service use the guidelines to inform care?

1. No
2. Yes
3. Partially

**If** P20.1 = ‘1’ OR ‘2’

- 1. : How relevant do you think they are for the care of kidney patients?

1. Not Relevant
2. Somewhat Relevant
3. Very Relevant
4. : Please specify any other national or professional guidelines, including COVID-19 guidelines that inform psychosocial care

[FREE TEXT]

**If text entered:**

- 1. : How useful were these guidelines?

1. Not Useful
2. Somewhat useful
3. Very useful
4. : What types of psychological treatments and interventions are offered

| 1. Cognitive Behavioural Therapy |  |
| --- | --- |
| 1. Acceptance and Commitment Therapy |  |
| 1. Compassion-focused therapy |  |
| 1. Behavioural therapy |  |
| 1. Interpersonal therapy |  |
| 1. Behaviour change interventions |  |
| 1. Counselling |  |
| 1. Art therapy |  |
| 1. Other |  |

**If P22 = ‘7’**

**P22b:** Please Specify

**[FREE TEXT]**

P23 : How are treatments **delivered (tick all that apply)**

|  | Usually | During Covid |
| --- | --- | --- |
| 1. Individually |  |  |
| 1. Group |  |  |
| 1. Family / carer |  |  |
| 1. Other |  |  |

**If P23 = ‘4’**

**P23b:** Please specify

**[FREE TEXT]**

**If P23 = ‘1’**

P24: What modes of treatment delivery do you use for **Individuals** **(tick all that apply)**

|  | Usually | During Covid |
| --- | --- | --- |
| 1. in person |  |  |
| 1. telephone |  |  |
| 1. Video conference (teams / zoom etc) |  |  |
| 1. Digital provision (e.g. apps) |  |  |
| 1. Other |  |  |

**If P24 = ‘5’**

**P24b:** Please specify

**[FREE TEXT]**

**If P23 = ‘2’**

1. : What modes of treatment delivery do you use for Groups (tick all that apply)

|  | Usually | During Covid |
| --- | --- | --- |
| 1. in person |  |  |
| 1. telephone |  |  |
| 1. Video conference (teams / zoom etc) |  |  |
| 1. Digital provision (e.g. apps) |  |  |
| 1. Other |  |  |

**If P25 = ‘5’**

**P25b:** Please specify

**[FREE TEXT]**

**If P23 = ‘3’**

1. : What modes of treatment delivery do you use for Family/carer (tick all that apply)

|  | Usually | During Covid |
| --- | --- | --- |
| 1. in person |  |  |
| 1. telephone |  |  |
| 1. Video conference (teams / zoom etc) |  |  |
| 1. Digital provision (e.g. apps) |  |  |
| 1. Other |  |  |

**If P26 = ‘5’**

**P26b:** Please specify

**[FREE TEXT]**

1. : If in your opinion a patient might benefit from medication (e.g. an anti-depressant) who would normally consider this and then prescribe if appropriate? (tick all that apply)

| 1. Nephrologists |  |
| --- | --- |
| 1. Mental Health Services |  |
| 1. GPs |  |
| 1. Other members of the MDT (e.g. Nurse/ or pharmacy prescriber) |  |
| 1. No, we do not discuss the use of psychotropic medications |  |

1. : What challenges linked to living with kidney disease does your service support and by whom typically? (tick all that apply)

|  | Psychologist | Therapist | Counsellor | Psychiatrist | Other (Please specify) |
| --- | --- | --- | --- | --- | --- |
| 1. Adjustment |  |  |  |  | [FREE TEXT] |
| 1. Treatment decisions |  |  |  |  | [FREE TEXT] |
| 1. Mental Health Conditions |  |  |  |  | [FREE TEXT] |
| 1. Bereavement | ☐ | ☐ | ☐ | ☐ | [FREE TEXT] |
| 1. Treatment non-adherence |  |  |  |  | [FREE TEXT] |
| 1. Pain Management |  |  |  |  | [FREE TEXT] |
| 1. Fatigue Management |  |  |  |  | [FREE TEXT] |
| 1. Support for Kidney donors |  |  |  |  | [FREE TEXT] |
| 1. Management of comorbidities |  |  |  |  | [FREE TEXT] |
| 1. Late presenters |  |  |  |  | [FREE TEXT] |
| 1. End of life care support |  |  |  |  |  |
| 1. Other (please specify) |  |  |  |  | [FREE TEXT] |

**If P28 = ‘11’**

**P28b :** Please specify what **other challenges** you support

**[FREE TEXT]**

**P28b.1**

**P28b.2**

**P28b.3**

1. : Do you evaluate the psychosocial service your unit offers?
2. No
3. Yes
4. Don’t know

**If P29 =1**

- 1. : How do you evaluate the psychosocial service your unit offers (tick all that apply)?

| 1. Aggregated patient reported symptom outcome measures |  |
| --- | --- |
| 1. Aggregated patient reported functional outcome measures |  |
| 1. Aggregated patient reported experience questionnaires |  |
| 1. Aggregated patient reported global improvement rating |  |
| 1. Aggregated clinician reported global improvement rating |  |
| 1. Service audit metrics (patient throughput / resource use / numbers discharged) |  |
| 1. Other |  |

**If P29.1 = ‘7’**

**P29.1b:** Please specify

**[FREE TEXT]**

1. : Do you evaluate the impact of psychological treatment on individual patients?

0 No

1 Yes

98 Don’t know

**If P30.2 = ‘ 2**

P30.1: How do you evaluate the impact of psychological treatment on individual patients (tick all that apply)?

| 1. Patient reported symptom outcome measures |  |
| --- | --- |
| 1. Patient reported functional outcome measures |  |
| 1. Patient reported experience questionnaires |  |
| 1. Patient reported global improvement rating |  |
| 1. Clinician reported global improvement rating |  |
| 1. CORE outcome measure (34 item) |  |
| 1. Qualitative feedback forms |  |
| 1. Other |  |

**If P30.3 = ‘8’**

**P30.1b:** Please specify

**[FREE TEXT]**

Thank you for your time.

If you have any queries, please contact [kidneyresearch@herts.ac.uk](mailto:kidneyresearch@herts.ac.uk).

We will follow up with centres in due course.

THANK AND CLOSE

## **Three: Social Work Module**

**TO BE ANSWERED BY THE SOCIAL WORKER AS IDENTIFIED IN THE INSTRUCTION SECTION**

1. : How are your social worker staff **funded** and **managed? (Please tick all that apply)**

|  | Funded by… | Managed by |
| --- | --- | --- |
| 1. The Local Authority/Council |  |  |
| 1. The NHS trust |  |  |
| 1. Charity |  |  |
| 1. Other |  |  |

**If S1 = ‘4’ Funded by**

**S1b:** Please specify **Other** funder

**[FREE TEXT]**

**If S1 = ‘4’ Managed by**

**S1c:** Please specify **Other** manager

**[FREE TEXT]**

1. : Is the social work provision **dedicated** to the renal service?
2. No
3. Yes
4. Don’t know
5. Where are your renal social worker/s **based? (Tick all that apply)**

| 1. Outpatient’s clinics |  |
| --- | --- |
| 1. Ward based |  |
| 1. Hospital office based |  |
| 1. Local Authority/ Council offices |  |
| 1. Other |  |

**If S3 = ‘5’**

**S3b:** Please specify **location**

**[FREE TEXT]**

1. **:**Which are the **MAIN** patient groups to access your service, usually and during **Covid? (TATA)**

|  | Normally | During Covid |
| --- | --- | --- |
| 1. Inpatient nephrology |  |  |
| 1. Transition (young to adult clinics) |  |  |
| 1. Mild to moderate CKD |  |  |
| 1. Advanced kidney care / low clearance clinic (Stage 3b-5) |  |  |
| 1. Immunosuppressed individuals (GN, vasculitis etc) |  |  |
| 1. In centre haemodialysis |  |  |
| 1. Satellite haemodialysis |  |  |
| 1. Home dialysis (HD and PD) |  |  |
| 1. Assisted PD |  |  |
| 1. Acute Transplant surgery |  |  |
| 1. Transplant follow up |  |  |
| 1. Living donation |  |  |
| 1. Conservative / Supportive care |  |  |
| 1. End of life care |  |  |

1. : Where do you get **most** of your social work referrals from; **please give approximate proportions of how many are in-patients or out-patients?**
2. In-patients **[FREE TEXT]**%
3. Out-patients [**FREE TEXT]**%
4. : What are the **MAIN** referral routes into your service for assessment or treatment? **(tick all that apply)**

|  | Normally | During Covid |
| --- | --- | --- |
| 1. From the medical or surgical team |  |  |
| 1. From the MDT |  |  |
| 1. Hospital social work team |  |  |
| 1. Family/carers |  |  |
| 1. Via proactive screening for Social difficulties |  |  |
| 1. Self-referral |  |  |
| 1. Via external agencies (for example KCUK, carer support services) |  |  |
| 1. Community social work services |  |  |
| 1. GP |  |  |
| 1. Psychological practitioners |  |  |
| 1. Relatives/carers |  |  |
| 1. Other |  |  |

**If S6 = ‘12’**

**S6b:** Please specify **Other** pathway

**[FREE TEXT]**

1. : Do you have a **formal waiting list** for your social work service?
2. No
3. Yes
4. Don’t know
5. : On average, how long do patients wait to be seen as a **new referral? (Please state usually and during covid) (if there is no change please tick the same for both)**

|  | Usually | During covid |
| --- | --- | --- |
| 0-2 weeks | ☐ | ☐ |
| 2-4 weeks | ☐ | ☐ |
| 1 month+ | ☐ | ☐ |

1. : Do you provide any **information / support** to people who are waiting to be seen?
2. No
3. Yes

**If S9 = ‘2 yes, please specify what this might typically be, and how it is provided**

**S9b:** Please specify what **information/support** is given and how it is provided

**[FREE TEXT]**

1. : Which **national** or **professional guidelines** most inform your social work practice

| 1. | **[FREE TEXT]** |
| --- | --- |
| 2. | **[FREE TEXT]** |
| 3. | **[FREE TEXT]** |
| 4. | **[FREE TEXT]** |
| 5. | **[FREE TEXT]** |

- 1. **:** Please indicate how **useful** you found these guidelines for the care of kidney patients:

|  | Not useful | Somewhat useful | Very useful |
| --- | --- | --- | --- |
| REDCap to pull through list from S10 |  |  |  |
|  |  |  |  |

1. : What is the **key role** of the social worker in the delivery of social care to **patients and carers?** **(tick all that apply)**

| 1. Identification of need |  |
| --- | --- |
| 1. Referral to appropriate agency |  |
| 1. Low level support (blue badge application, completing forms) |  |
| 1. Welfare/benefits advice and support to complete assessments |  |
| 1. Informal Counselling |  |
| 1. Information provision |  |
| 1. Low level emotional support - Reassurance & Empathy |  |
| 1. Carer bereavement support |  |
| 1. care needs assessment, co-ordination and follow |  |
| 1. Grant applications (financial) e.g. KCUK |  |
| 1. Pre-dialysis assessment and education |  |
| 1. Conservative management (liaison community services/joint visits community service |  |
| 1. Supportive/Palliative Care (Dialysis & Transplant patients) |  |
| 1. Carer Assessments |  |
| 1. Care assessments |  |
| 1. Discharge planning |  |
| 1. Other |  |

**If S11 = ‘18’**

**S11b:** Please specify **Other** key roles

1. **[FREE TEXT]**
2. **[FREE TEXT]**
3. **[FREE TEXT]**
4. : Of the **key roles** the social worker fulfils in the delivery of social care to patients and carers, please identify those which saw the greatest **increase / decrease** in demand during Covid? **(Increase / Decrease up to 3 each)**

| Increase |  |
| --- | --- |
| 1. | **REDCap to pull through drop down list from those above** |
| 2. |  |
| 3. |  |
| Decrease |  |
| 1. |  |
| 2. |  |
| 3. |  |

1. : Which **internal** and **external services** do you tend to engage with or refer patients to for additional psychosocial support: **(tick all that apply and identify for which groups, patients and/or carers)**

|  | Patients | Carers |
| --- | --- | --- |
| 1. IAPT |  |  |
| 1. Palliative care (community/hospital) |  |  |
| 1. Community Mental health services |  |  |
| 1. Adult Care Services |  |  |
| 1. Citizens advice Bureau |  |  |
| 1. Psychiatry |  |  |
| 1. Primary care – GPs |  |  |
| 1. Psychology services |  |  |
| 1. Counselling |  |  |
| 1. Self-help online services |  |  |
| 1. KCUK online counselling service |  |  |
| 1. Welfare officer |  |  |
| 1. Youth Workers |  |  |
| 1. Money Advice Unit (MAU) |  |  |
| 1. Carers UK |  |  |
| 1. Age UK |  |  |
| 1. CRUSE (bereavement service) |  |  |
| 1. Chaplain / Spiritual Care services |  |  |
| 1. Other |  |  |

**If S13 = ‘19’**

**S13b:** Please specify **Other** services

**[FREE TEXT]**

1. : Did **access** to internal and external psychosocial support services change during Covid? **Please tick all that apply**

|  | Access improved [1] | Access stayed the same [2] | Access got worse [3] | Don’t know / not applicable [98] |
| --- | --- | --- | --- | --- |
| RedCap to pull through indicated list from S14 [s14] = ‘1’ |  |  |  |  |
| [S14] = ‘2’ |  |  |  |  |
|  |  |  |  |  |

1. : During **Covid** which areas (including social issues and access to services) have increased in difficulty or complexity? **(Please tick all that apply)**

| 1. Mental health |  |
| --- | --- |
| 1. Alcohol advisory |  |
| 1. IAPT |  |
| 1. Social services |  |
| 1. GP |  |
| 1. Welfare benefits |  |
| 1. Housing |  |
| 1. Employment support |  |
| 1. Community groups |  |
| 1. Adaptations/equipment (bathroom/stair rails/general repairs/ |  |
| 1. Environmental concerns (hoarding issues/cleaning) |  |
| 1. Other |  |

**If S15 = ‘12’**

**S15b:** Please specify **Other** areas

**[FREE TEXT]**

1. : How are **interventions** delivered **(please tick all that apply)**

|  | Usually | During Covid |
| --- | --- | --- |
| 1. Individually |  |  |
| 1. Group, |  |  |
| 1. Family / carer |  |  |
| 1. Other |  |  |

**If S16 = ‘4’**

**S16b:** Please specify

**[free text]**

**If S16 = ‘1’**

1. : What modes of treatment delivery do you use for Individuals (tick all that apply)

|  | Usually | During Covid |
| --- | --- | --- |
| 1. in person |  |  |
| 1. telephone |  |  |
| 1. Video conference (teams / zoom etc) |  |  |
| 1. Digital provision (e.g. apps) |  |  |
| 1. Other |  |  |

**If S17 = ‘5’**

**S17b:** Please specify **Other** delivery mode

**[FREE TEXT]**

**If S16 = ‘2’**

1. : What modes of treatment delivery do you use for Groups (tick all that apply)

|  | Usually | During Covid |
| --- | --- | --- |
| 1. in person |  |  |
| 1. telephone |  |  |
| 1. Video conference (teams / zoom etc) |  |  |
| 1. Digital provision (e.g. apps) |  |  |
| 1. Other |  |  |

**If S18 = ‘5’**

**S18b:** Please specify **Other** delivery mode

**[FREE TEXT]**

**If S16 = ‘3’**

1. : What modes of treatment delivery do you use for Family/Carer (tick all that apply)

|  | Usually | During Covid |
| --- | --- | --- |
| 1. in person |  |  |
| 1. telephone |  |  |
| 1. Video conference (teams / zoom etc) |  |  |
| 1. Digital provision (e.g. apps) |  |  |
| 1. Other |  |  |

**If S19 = ‘5’**

**S19b:** Please specify **Other** delivery mode

**[FREE TEXT]**

1. : Approximately how many **NEW patients** does your service encounter per month?

**[FREE TEXT]**

1. : Approximately how many **FOLLOW-UP** patients does your service encounter per month?

**[FREE TEXT]**

1. : **Broadly,** how has this changed during **COVID?**
2. No change
3. Increase in caseloads
4. Decrease in caseloads
5. : From your perspective what is the **main gap** in psychosocial provision in your service? **[FREE TEXT]**

Thank you for your time. If you have any queries please contact [kidneyresearch@herts.ac.uk](mailto:kidneyresearch@herts.ac.uk).

We will follow up with centres in due course.

THANK AND CLOSE
